# Chamber‐by‐Chamber Measurements of Planktonic Foraminiferal Mg, Sr, and Na to Ca Ratios With Femtosecond LA‐ICP‐MS

**DOI:** 10.1002/rcm.70026

**Published:** 2026-01-19

**Authors:** Toshihiro Yoshimura, Qing Chang, Naohiko Ohkouchi, Yoshiyuki Ishitani, Dana Ulanova, Hirotoshi Endo, Junichiro Kuroda, Yurika Ujiié

**Affiliations:** ^1^ Biogeochemistry Research Center Japan Agency for Marine‐Earth Science and Technology (JAMSTEC) Yokosuka Japan; ^2^ Volcanoes and Earth's Interior Research Center Japan Agency for Marine‐Earth Science and Technology (JAMSTEC) Yokosuka Japan; ^3^ Institute for Extra‐Cutting‐Edge Science and Technology Avant‐Garde Research Japan Agency for Marine‐Earth Science and Technology (JAMSTEC) Yokosuka Japan; ^4^ Faculty of Agriculture and Marine Science Kochi University Nankoku Kochi Japan; ^5^ Marine Core Research Institute Kochi University Nankoku Kochi Japan; ^6^ National Institute of Technology Tsuruoka College Tsuruoka Yamagata Japan; ^7^ Atmosphere and Ocean Research Institute The University of Tokyo Kashiwa Chiba Japan

**Keywords:** chamber, femtosecond, foraminifera, LA‐ICP‐MS, Mg/Ca, temperature

## Abstract

**Rationale:**

Distribution patterns of foraminifera are controlled by environmental parameters such as temperature, salinity, and nutrient concentrations in each water mass. Since trace elements to Ca ratios of marine microfossil calcite test of foraminifera record environmental and ecological habitat information, we used femtosecond (fs) LA‐ICP‐MS to obtain accurate chamber‐by‐chamber Mg/Ca, Sr/Ca, and Na/Ca of four foraminifera species to clarify the impact of foraminiferal depth migration on paleoceanographic reconstruction. The Mg/Ca and Sr/Ca ratios were measured with precision better than 5%, fulfilling the accuracy typically required for paleoceanographic reconstructions. We also examined the differences in element ratios due to the pretreatment cleaning methods for extracting accurate paleoceanographic information.

**Methods:**

The fsLA‐ICP‐MS has the advantage of less matrix and instrumental element fractionation effects on elements with high condensation temperatures. We also applied the use of multiple carbonate standard materials for concentration standardization.

**Results:**

The fsLA‐ICP‐MS analysis was optimized using a spot size of 30 μm or larger with a laser repetition frequency of 5 to 15 Hz in a circular analytical trajectory. A comparison between ultrasonic and oxidative cleaning protocols revealed that oxidative test cleaning with perchloric acid and hydrogen peroxide achieved higher reproducibility and more efficient impurity removal compared to ultrasonic cleaning with ultrapure water and methanol. Repeated analysis on the same chambers of two species, *O. universa* and *P. obliquiloculata*, yielded mean relative standard deviations for Mg/Ca and Sr/Ca of < 5%.

**Conclusions:**

A quantitative method was rapidly developed for determination of Mg, Sr, and Na to ratios of biogenic carbonates of foraminifera. *T. sacculifer* showed no chamber‐by‐chamber Mg/Ca variation in calcifying temperature, but average test Mg/Ca temperature decreased by 1.4°C with the addition of the final sac‐like chamber and final calcite layer. *G. menardii* showed a ~7°C difference among chambers suggesting upward migration in the shallow part of the thermocline.

**Abbreviations**
DIC: dissolved inorganic carbon
fs: femtosecond
LA‐ICP‐MS: laser ablation‐inductively coupled plasma‐mass spectrometry
MeOH: methanol
PFA: perfluoroalkoxy alkane
RSD: relative standard deviation
RV: research vessel
TE: Trace elements
UPW: ultrapure water
WOA: World Ocean Atlas
WPWP: Western Pacific Warm Pool

## 1 Introduction

Planktonic foraminifera tests have been widely used in paleoceanographic reconstruction because they show species‐specific distributions according to water masses and habitat depths and different seasonal reproductions as shown by direct observations of plankton nets and sediment traps [1, 2, 3, 4]. Trace elements to calcium (Ca) ratios (TE/Ca, e.g., Mg/Ca, Sr/Ca, and Na/Ca) of planktonic foraminiferal tests reflect habitat environmental parameters, such as water temperature, salinity, and nutrient concentrations [5, 6, 7, 8, 9, 10, 11, 12]. Since the distribution pattern of planktonic foraminifera is associated with variable physicochemical conditions in each of the water masses and ocean basins, the equation for TE/Ca proxies must be carefully calibrated for each survey area.

The TE/Ca of foraminifera tests are usually measured by using bulk samples, which homogenize a few to tens of individuals. The general assumption of bulk analysis of multiple foraminifera is that the bulk TE/Ca provides a snapshot of the marine environment equivalent to several weeks, integrating the entire life history of the individual, while the measurement at the individual level shows intra‐ and inter‐population variation reflecting seasonality and life history [13, 14, 15]. Indeed, chamber‐by‐chamber analyses of foraminiferal Mg/Ca, δ^18^O, and δ^13^C have succeeded to estimate water temperature at each chamber‐formation (calcification) depth during individual life cycles, e.g., [14, 16]. In accordance with the principle of the Mg/Ca thermometer, in which the endothermic reaction of Mg replacing Ca is promoted at higher temperatures, the relationship equations have been calibrated for each foraminifer species and applied to foraminiferal species in sediments [17, 18] (see Supplementary text for foraminifera ecology and element incorporation into tests). The results of *K*‐edge XANES analysis of Mg in the foraminifera test indicate that the majority of Mg chemical species are structurally substituted Mg components [19, 20]. These characteristics of the chemical form support the mechanism of the Mg/Ca thermometer based on the endothermic reaction of Mg substituting for Ca [19]. Recently, analysis of individual foraminifera tests has also been used to reconstruct past changes in seasonal and annual temperature fluctuations [21]. The approach of focusing on the bulk and chamber‐specific composition of single individuals reveals the relationship between the chemical composition of tests and high‐time‐resolution oceanographic conditions, as well as inherent ecological biases.

As the calibration of precise paleoceanographic proxies advances, the influence of biological mechanisms on intra‐shell chemical composition has been verified through various micro‐analytical approaches. At the fine submicron spatial resolution, electron microprobe analysis highlighted the presence of Mg concentration bands within the shell, suggesting they reflect diurnal variations in the biological activity of algal symbionts and host foraminifera [22]. Subsequent SIMS analysis further contributed significantly to elucidating the biomineralization mechanism, revealing the presence of synchronized banding patterns involving multiple elements [23] and the specific enrichment of Mg and Na within the organic membrane template [24].

The accuracy of microscale analysis of such element compositions has been tested and compared with bulk analyses [13]. Laser ablation inductively coupled plasma mass spectrometry (LA‐ICP‐MS) has been applied as a quantitative and rapid microanalysis technique for measuring TE/Ca in planktonic foraminifera shells [22, 25, 26, 27, 28, 29, 30, 31, 32, 33, 34, 35]. A deep UV excimer LA‐ICP‐MS has been used to generate TE/Ca profiles at micron‐ to submicron resolution through the walls of foraminifera tests and to identify and remove diagenetic components formed after accumulation of foraminiferal tests in the sediment column [27, 36, 37]. The femtosecond LA‐ICP‐MS (fsLA‐ICP‐MS) has the advantage of less matrix and instrumental element fractionation effects on elements with high condensation temperatures, including Ca, and is also unlikely to cause laser‐induced isotopic fractionation [38, 39, 40, 41]. The use of fsLA‐ICP‐MS is potentially a new paleoceanographic application aiming for better accuracy and precision [35]. Here, the measurement precision (1 relative standard deviation percentage) of TE/Ca data has been determined through repeated measurements of calcium carbonate materials, and measurement accuracy has been evaluated using published Mg/Ca reference values from international reference materials such as JCt‐1, JCp‐1, NIST SRM 610, and MACS‐3, which possess different TE/Ca ratios [35]. It has recently been shown that in measurements of carbonate standards, a high concentration linearity can be achieved even when using standardization with very different physical properties, such as silicate glasses [35]. Therefore, the application of high accuracy fsLA‐ICP‐MS method will lead to accurate temperature reconstruction, as it is less likely to cause concentration bias between solution and laser methods even when using a Mg/Ca thermometer equation mostly calibrated by solution analysis. Intra‐laboratory reproducibility of foraminiferal Mg/Ca ratios measured by solution analysis using a consistent washing protocol has been reported to achieve less than ±2% [42], and the temperature dependence of Mg/Ca thermometer is ~3% per 1°C [42, 43]. This temperature sensitivity represents the reconstruction of temperature changes associated with the migration of foraminifera habitat depth within a water column (Figure S1). For practical purposes, we aimed accuracy of a few percent to enable the functional determination of habitat depth of foraminifera within water masses.

In addition, the pre‐cleaning protocol using oxidizing reagents prior to solution Mg/Ca analysis [42] differs from that used in LA‐ICP‐MS analysis: deionized water and methanol cleaning followed by pre‐ablation cleaning for surface contaminants [14, 37]. Cleaning with deionized water and methanol is highly effective for preparing foraminifera extracted from geological samples [31, 37]. Residual shell‐surface contaminants can subsequently be removed by TE/Ca depth profiling [34, 35]. To further evaluate the extent to which the measured element ratios caused by differences in protocol can be a source of bias in analytical values and between‐ and within‐individual variability, we report on the effects of LA‐ICP‐MS analysis of tests that have undergone both oxidative reagent cleaning and methanol cleaning for solution analysis. This strategy is intended to bridge the gap between solution analysis and laser analysis methods. Recently, the Na/Ca paleoceanographic proxy has drawn attention [33, 44, 45], and alkaline reagents replacing NaOH, traditionally used in cleaning reagents for sedimentary foraminifera, have been proposed [45]. Cleaning solution analysis must be evaluated not only for its ability to effectively remove shell surface deposits but also for its potential to cause TE/Ca offset, including potential carryover from reagents. Therefore, we also investigate the elemental leaching behavior into the supernatant using the conventional NaOH method and the NH_4_OH [45, 46] and KOH (this study) methods.

In this study, we aimed to address (1) optimization of fsLA‐ICP‐MS analysis by applying a standard sample with a matched matrix, and evaluation of the impact of chemical cleaning of foraminifera on TE/Ca; (2) chamber‐ and species‐specific incorporation of Mg, Sr, and Na incorporation into foraminifera calcite tests composed of additional‐growth chambers. Four foraminifera species picked from surface sediments in the Western Pacific Warm Pool (WPWP) with potential chemical compositional shifts associated with chamber addition (*Trilobatus sacculifer*, *Globorotalia menardi*) and species with homogeneous final chambers or calcite layers, useful for method validation (*Orubulina universa*, *Pulleniatina obliquiloculata*), were selected for analysis on an inter‐chamber and individual basis by using the fsLA‐ICP‐MS. Since the factors causing deviations in paleoceanographic data are thought to be due to the preservation/cleaning conditions of the tests and ecological differences at the individual level, we aimed to establish a more robust geochemical approach by applying chamber‐specific analysis.

## 2 Material and Methods

### 2.1 Oceanographic Setting and Depth Migration of Foraminifera

The WPWP is a large water mass characterized by a high sea surface temperature (SST) of > 28°C with a deep thermocline (Figure S1). The WPWP is an important global climate feature, supplying heat and moisture to high latitudes through ocean currents and atmospheric systems, and an understanding of how this feature has changed over time is central to our understanding of long‐term climate dynamics. Paleoceanographic reconstructions from this region allow us to quantify temporal and spatial changes in the physicochemical changes of this region and understand long‐term changes in El Niño‐Southern Oscillation variability, thermocline structure, etc., e.g., [47, 48].

*T. sacculifer* and *Globigerinoides ruber* are representative species of the oligotrophic water mass of the WPWP, with both species, as well as *P. obliquiloculata*, comprising more than 10% of the total foraminifera flux to the sediment in the West Caroline Basin [2]. Examination of δ^18^O and Mg/Ca temperature data in surface sediment samples from the tropic Atlantic and Pacific oceans indicates that *G. ruber* records SST, whereas *T. sacculifer* records temperature at slightly deeper depths of 20–30 m in the mixed layer [9, 49]. In the WPWP at the Manihiki Plateau region, apparent calcification depths based on Mg/Ca indicated that *G. ruber* was the shallowest species, followed by *T. sacculifer* and *P. obliquiloculata* with increasing depth [4]. *G. menardii* is known as an ontogenetic vertical migrating species with a slightly deeper habitat depth than *P. obliquiloculata*, e.g., [50]. It is of interest for paleoceanography reconstruction to determine whether the characteristics of the calcification depth of these species are recorded in the chemical composition of each chamber at the individual level. In addition, *T. sacculifer* exhibits two morphologically distinct forms with paleoceanographic implications, distinguished by the presence or absence of a sac‐like chamber attached to its outermost layer. The sac‐like chamber in *T. sacculifer* forms approximately 24 to 48 h prior to gamete release as a sign indicating imminent gamete formation [51]. While this sac‐like chamber is a clear indicator of impending gamete formation, it is not an essential structure. The frequency of sac‐like chamber formation increases with increased feeding frequency and food supply. These were measured separately to account for differences in habitat depth.

Population dynamics of *G. menardii* indicates that large specimens migrate to shallow depths; then released gametes sink to deeper waters, e.g., [52]. Recent plankton net study in the subtropical Atlantic Ocean, with contrasting observations of mature specimens sinking and then gametes rising, suggests that patterns of ontogenetic vertical migration vary by region and ecological conditions [53]. Both *P. obliquiloculata* and *G. menardii* appear with abundant phytoplankton and high productivity associated with upwelling currents, e.g., [54]. The species‐specific differences in habitat depth allow reconstruction of water mass structure, e.g., Mg/Ca differences between surface and thermocline species (e.g., *G. ruber* and *P. obliquiloculata*) can be used to track changes in upper water column stratification [30, 55]. As sediments from the WPWP region are of critical importance for paleoenvironmental reconstruction of tropical water masses as those described above, it is important to understand how foraminifera ecology is linked to water mass features, such as thermocline, and to identify the causes of variation in test chemical composition.

### 2.2 Foraminifera Samples

A pilot‐core sediment was obtained from a site in the Ontong Java Plateau (Site MR1402‐PL04; 2°03.00′ N, 156°06.48′ E, water depth 2447 m) in the western equatorial Pacific during the *RV* Mirai cruise MR14‐02. The major lithology of the sediment is light gray to light olive gray calcareous ooze with siliceous fossils. Samples were obtained from two layers: 0–5.0 and 5.0–8.5 cm in the pilot core, and the age of the sediment at a depth of 8.5 cm from the surface is estimated to be ~5000 years ago according to the sedimentation rates in this region [56]. Sediment samples were washed through a 63 μm sieve and dried at 65°C at least overnight. From them, we picked four species, *T. sacculifer* (with and without sac‐like final chamber), *G. menardii*, *P. obliquiloculata*, and *O. universa* under a stereomicroscope. The first three species are representative of tropical water mass and the last one species is optimal to examine the reproducibility of TE/Ca measurements using multiple analysis points, because its spherical chamber completely envelopes all earlier chambers. In each specimen of *T. sacculifer*, *G. menardii*, and *P. obliquiloculata*, we identified chambers and gave a number from final (latest formed) chamber to younger chambers as F0 to F3.

Reproducibility of the element ratio analysis was confirmed by triplicate measurements on 10 individuals of *O. universa* as well as three individuals of *P. obliquiloculata*, whose final chambers and final smooth calcite were easily identifiable. We note that the *O. universa* and *P. obliquiloculata* experiments are only intended to examine analytical reproducibility within the chamber, as individual differences in their ecological history should affect inter‐individual TE/Ca variation.

### 2.3 Preparation of Foraminiferal Tests for TE/Ca Measurements

Prior to TE/Ca measurements, foraminiferal tests were cleaned by removing other calcareous materials, such as calcareous nannofossils, clays, and authigenic precipitates, which are attached to the test surface and are contained inside the test. In this study, two different cleaning methods were employed for fsLA‐ICP‐MS analysis. The first one was the removal of the attached materials by ultrasonication with methanol followed by rinsing with ultrapure water [31]. Multiple foraminiferal tests were placed in a 2 mL acid‐cleaned tube and 1 mL of MeOH was added. These tubes were floated in a 100‐cc beaker filled with water to avoid test damage during ultrasonication. Beakers were placed in the bath and ultrasonication with a frequency of 40 kHz was conducted. This process was repeated several times until all microscopically visible loose material (calcareous nannofossils) was removed.

The second cleaning method was modified from the oxidative chemical cleaning protocol first developed by Barker et al. [42], which uses 30% H_2_O_2_, 1 M NaOH, 1% HClO_4_, and ultrasonication [57]. In this study, we diluted the reagents twice and used KOH instead of NaOH to avoid the effect of added sodium on Na/Ca measurement. Samples were submerged in a mixture of equal parts by volume of 15% H_2_O_2_ and 0.5 M KOH, and two consecutive 5‐min sessions of ultrasonication were conducted. Samples were then rinsed once in a mixture of equal parts by volume of 15% H_2_O_2_ and 0.5% HClO_4_ to remove organic matter (hereafter, H_2_O_2_–HClO_4_ cleaning). After drying in a clean bench, the TE/Ca (Mg/Ca, Sr/Ca, and Na/Ca) by femtosecond LA‐ICP‐MS were conducted for individuals whose chambers remained undamaged during chemical cleaning.

Furthermore, to verify the compatibility of this method with the conventionally used NaOH, bulk sediments from the same stratigraphic level as the samples were fractionated into the size fractions 63–150 and 150–250 μm, frequently used in paleoceanographic studies. Cleaning experiments were conducted using three types of alkali reagents (NaOH, NH_4_OH, and KOH) by replacing only the alkali reagent in the aforementioned procedure for each fraction. At each step, the supernatant was collected and measured by ICP‐MS. The elution amounts and element ratios relative to calcium were quantified for carbonate constituent elements and impurity elements frequently found in clastic and authigenic contaminants (Ca, Mg, Sr, Ba, Al, Ti, Mn, Fe, and Pb). Sodium and K were excluded from the measurement targets because they were present as concentrated liquids in the supernatant.

### 2.4 Femtosecond LA‐ICP‐MS

Trace element ratios were analyzed using a quadrupole ICP‐MS (iCAP‐Q, Thermo Fisher Scientific) equipped with a fsLA system with a wavelength range of 200/266 nm [58, 59]. The objective was to measure the bulk TE/Ca of each chamber stably by laser irradiation with a circular trajectory. Therefore, the ablation rate of our fsLA system is like the previous study using the same analytical mode [59], and a direct comparison of ablation rate to other previously published systems, e.g., [34] would not be possible. The ablation sample aerosol transported by the helium gas stream is introduced into a perfluoroalkoxy alkane (PFA) buffer chamber with an internal volume of 150 mL [59]. There, it is mixed with argon make‐up gas and jointly injected into the ICP‐MS to achieve a stable signal. We also adopted a repetition rate of 5 Hz or higher, which is less sensitive to microstructural effects, although the spatial resolution (i.e., within‐wall element variation) is reduced [34]. This analytical strategy is different from the previous studies that have reported the depth profiles of TE/Ca corresponding to the test microstructure by laser analysis using a lower repetition rate (e.g., mainly 1–3 Hz) with cell devices that have rapid wash‐out times [28, 58, 59]. The strategy of this chamber‐specific bulk analysis was to obtain the average value for each chamber, even though the information on the element distribution of the cross section was lost, and the intention was to make a direct comparison with the conventional solution analysis and temperature conversion formula. In LA‐ICP‐MS analysis, the contribution of diagenesis and surface contaminants is determined from the element profile, e.g., [14, 37] but we avoided these effects by using oxidative cleaning, as similar to solution TE/Ca analysis [42].

Ablation of foraminiferal tests was performed in a small volume ablation cell (~20 cm^3^ internal volume) under laminar flow of helium carrier gas [58]. The wavelength of 266 nm was selected for all analyses. The laser pulse widths and laser fluences on the sample surface were < 300 fs and ~12 J cm^−2^, respectively [58]. The size and shape of the laser ablation spot was circular with a diameter of 40–60 μm (see photos of ablated samples in Figure S2). The ablated sample aerosol was transferred by He gas flow into a PFA buffer chamber with an internal volume of 150 mL, mixed with Ar make‐up gas, and introduced into the ICP‐MS. Wash‐out of the Ca signal took ~20 s, setting an overall cell washing time of at least 1 min for the measurement. The isotopes selected were ^23^Na, ^25^Mg, ^43^Ca, and ^86^Sr. For other detailed analysis conditions, please refer to the Supplementary Information.

The standard samples used to calibrate the element ratios were JCp‐1 from coral and JCt‐1 from the giant clam, both issued by the National Institute of Advanced Industrial Science and Technology (https://gbank.gsj.jp/geostandards/welcome.html), with consensus values of Mg/Ca for JCp‐1 and JCt‐1 of 4.199 ± 0.065 mmol/mol and 1.289 ± 0.045 mmol/mol, and Sr/Ca of 8.838 ± 0.042 mmol/mol and 1.680 ± 0.026 mmol/mol, respectively [60]. Since no joint comparison was performed for Na/Ca, we calculated the values based on the values of Okai et al. [61]. The difference in TE/Ca between Hathorne et al. and Okai et al. is roughly 2% [60, 61], which is one‐third to one‐eighth of the reproducibility of repeated analyses of the same test for Na/Ca (Table 1). Some previous studies have produced calibration curves using NIST glass reference materials, but the analytical accuracy of fsLA‐ICP‐MS on NIST glass and MACS3, JCp‐1 and JCt‐1 carbonates has been reported to be independent of the fractionation effect due to material difference [35]. Given the high linearity of the element ratios for fsLA‐ICP‐MS calibration is guaranteed with these international reference materials above [35], this study used JCp‐1 and JCt‐1, which have similar matrices to the foraminiferal test, to create a linear calibration curve for quantification. In terms of matrix matching for ICP‐MS analysis, it is recommended that the major element Ca should be matched between standards and samples in solution analysis, e.g., [60]. JCp‐1 and JCt‐1 are reference materials for which consensus element ratios have been reported in the field of paleoceanography [60], and are standard materials capable of providing multi‐point calibration for element ratios such as Mg/Ca and Sr/Ca. Furthermore, we added that using consensus values for LA‐ICP‐MS calibration is advantageous because it minimizes calibration discrepancies between solution analysis and instrument calibration.

**TABLE 1:** Results of triplicate measurements of the multiple individuals of the final spherical chamber of *Orbulina universa* and the final smooth calcite of *P. obliquiloculata*. Two different cleaning methods were applied for *O. universa* , and standard deviations (mmol/mol) and relative standard deviations (%) were noted together.

Depth	Species	*N*	Cleaning	Mg/Ca	SD	RSD	Sr/Ca	SD	RSD	Na/Ca	SD	RSD
cm			
mmol/mol		%	mmol/mol		%	mmol/mol		%
0.0–5.0	*P. obliquiloculata*‐#1	3	H_2_O_2_‐HClO_4_	3.39	0.16	4.6	1.39	0.01	0.9	7.90	1.28	16.2
0.0–5.0	*P. obliquiloculata*‐#2	3	H_2_O_2_–HClO_4_	2.13	0.05	2.1	1.34	0.10	7.6	6.58	0.56	8.6
0.0–5.0	*P. obliquiloculata*‐#3	3	H_2_O_2_–HClO_4_	2.42	0.08	3.4	1.41	0.02	1.5	7.43	0.46	6.1
0.0–5.0	*O. universa* ‐#1	3	H_2_O_2_–HClO_4_	4.73	0.18	3.9	1.35	0.04	3.0	7.54	0.12	1.6
0.0–5.0	*O. universa* ‐#2	3	UPW‐MeOH	6.38	0.30	4.7	1.41	0.03	2.4	7.53	0.40	5.3
0.0–5.0	*O. universa* ‐#3	3	UPW‐MeOH	10.46	1.36	13.0	1.45	0.03	1.9	10.42	1.08	10.4
0.0–5.0	*O. universa* ‐#4	3	UPW‐MeOH	12.55	1.04	8.3	1.47	0.03	1.8	11.57	1.45	12.5
5.0–8.5	*O. universa* ‐#5	3	H_2_O_2_–HClO_4_	7.14	0.44	6.1	1.52	0.07	4.5	11.91	1.97	16.6
5.0–8.5	*O. universa* ‐#6	3	H_2_O_2_–HClO_4_	8.90	0.33	3.7	1.49	0.04	2.4	9.49	1.22	12.9
5.0–8.5	*O. universa* ‐#7	3	H_2_O_2_–HClO_4_	5.07	0.20	3.9	1.37	0.05	3.8	9.86	0.76	7.7
5.0–8.5	*O. universa* ‐#8	3	UPW‐MeOH	7.16	0.33	4.6	1.44	0.04	2.8	8.80	0.39	4.4
5.0–8.5	*O. universa* ‐#9	3	UPW‐MeOH	7.64	0.56	7.3	1.46	0.09	6.2	12.43	0.16	1.3
5.0–8.5	*O. universa* ‐#10	3	UPW‐MeOH	6.14	0.42	6.9	1.42	0.06	3.9	7.78	0.84	10.7

Jochum et al. (2019) proposed that the determination of trace element concentrations using nano‐processed certified reference materials is preferable for LA‐ICP‐MS measurements [62]. However, since the relatively abundant constituent elements, Na, Mg, Sr, U, and Ba, in JCp‐1 and JCt‐1 fall within the uncertainty range of natural carbonates [62] and are not necessarily affected by nano‐processing, the original powder of JCp‐1 and JCt‐1 was compressed in a tablet molding machine to form a homogeneous smooth surface and fixed with carbon double‐sided tape so that the sample height was approximately the same as that of foraminiferal tests. For calibration curves, each standard sample is measured about 10 times repeatedly, and measurement points with high intensities for Fe (as well as Al), which may be derived from the stainless steel of the tablet mold, are excluded from the data.

## 3 Results and Discussion

### 3.1 fsLA‐ICP‐MS Analysis

**FIGURE 1:**
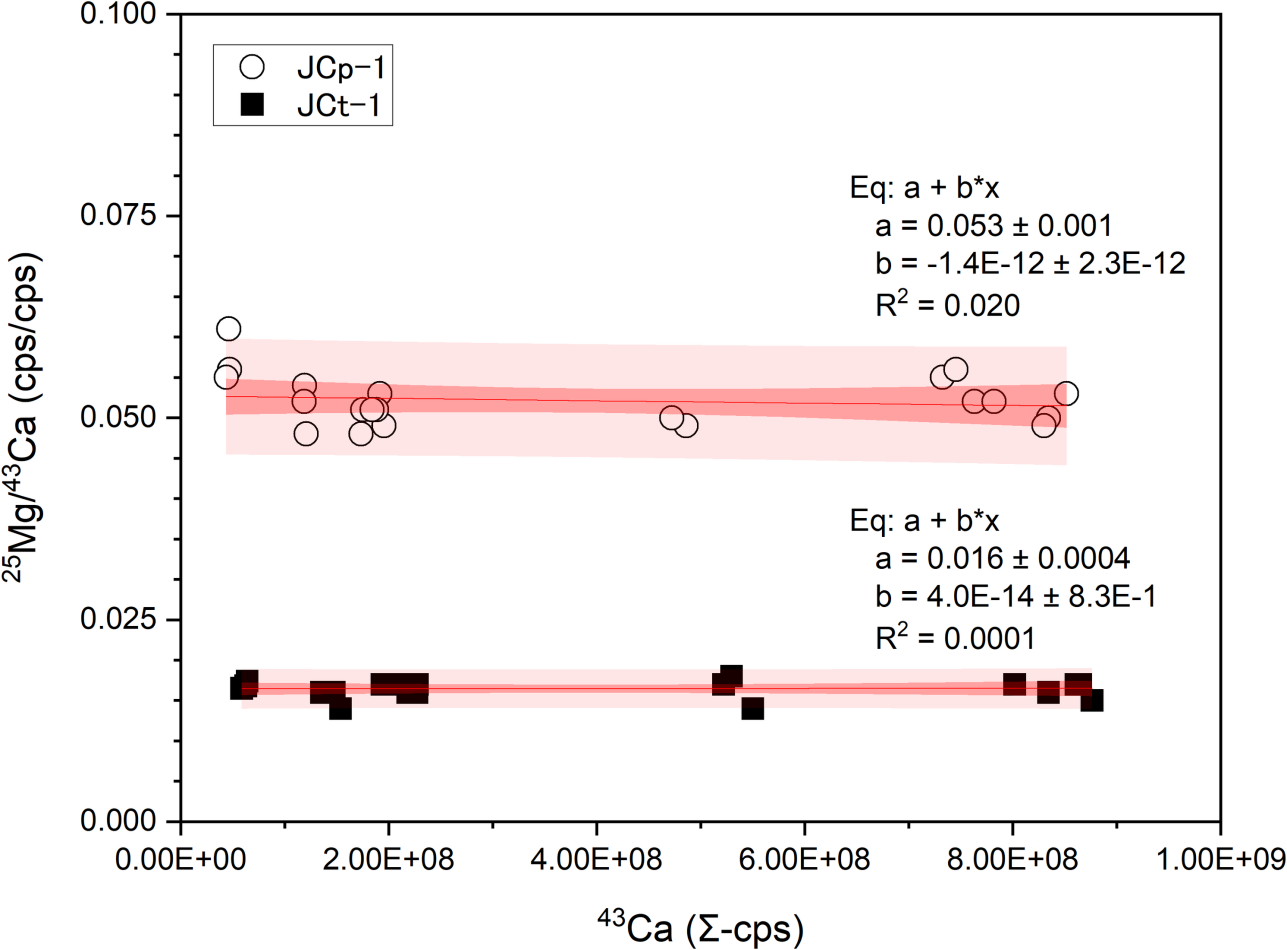
Sum of ^43^Ca signal intensities during LA‐ICP‐MS analysis versus ^25^Mg signal intensity relative to ^43^Ca. The analysis was performed using pelleted carbonate standards, JCp‐1 and JCt‐1, prepared from coral skeletons and giant clam shells, respectively. The ^43^Ca signal intensities were changed by varying the laser repetition frequency. Although the variation is large for very small sample introductions, there was no change in the element ratios with changes in the amount of Ca introduced, which has a lower ionization energy.

To optimize analytical parameters, we first examined the laser repetition frequency and the ablation trajectory, which are the same for foraminifera, using the JCp‐1. The laser repetition rate was changed from 1 to 10 Hz and the spot size from 30 to 70 μm (Figure 1). In this condition, the signal count ratio fluctuated by approximately one order of magnitude. As shown in Figure 1, a stable signal intensity ratio was obtained regardless of the amount of Ca introduced. In addition, only spot size and repetition rate were varied independently, but no specific condition caused a strong bias except for a tendency for repeatability to improve as the signal intensity increased.

**FIGURE 2:**
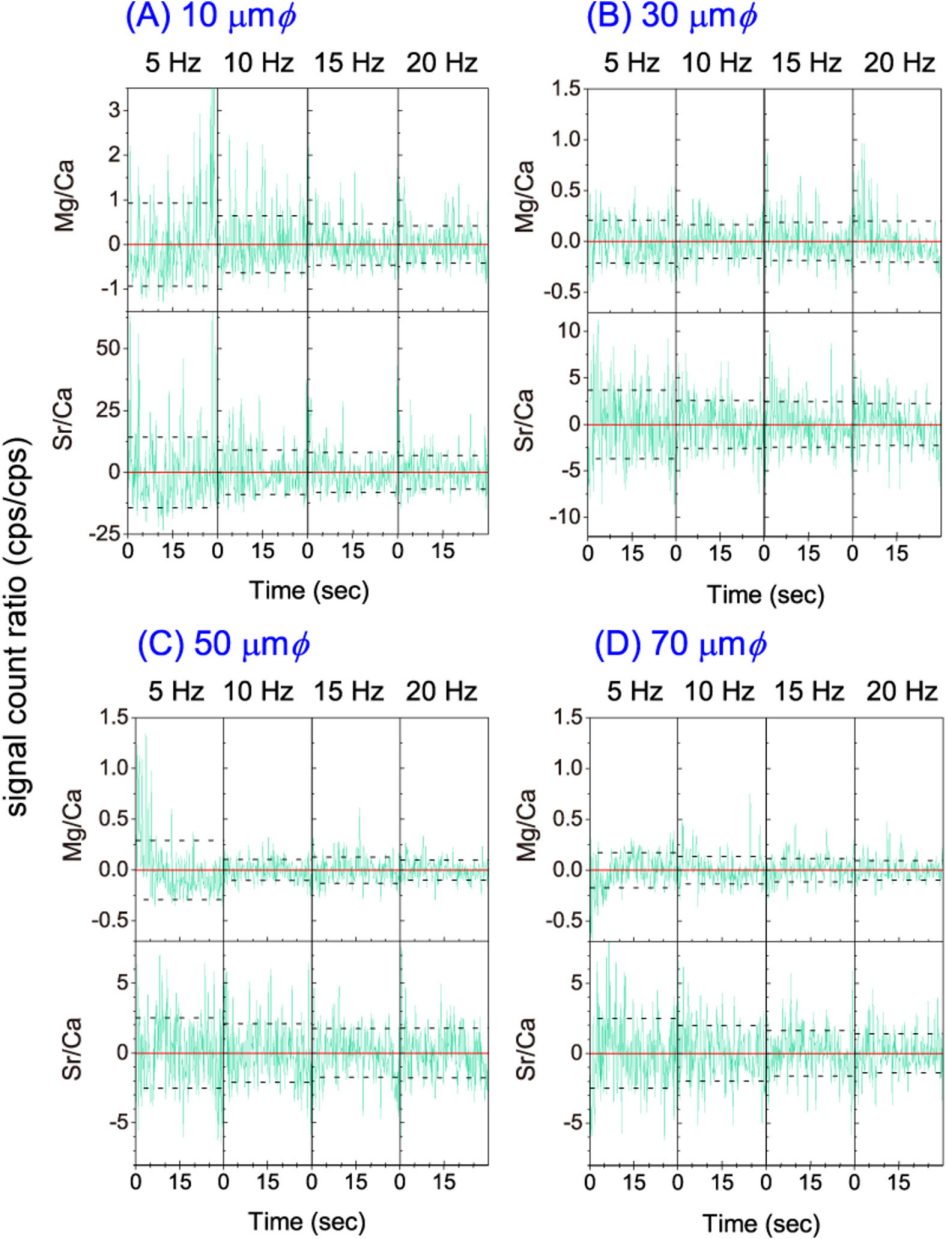
Stability of the signal intensity ratios (Mg/Ca and Sr/Ca) of JCp‐1 samples when the laser repetition frequency is changed to 5, 10, 15, and 20 Hz and the laser diameter is changed to 10, 30, 50, and 70 μm (see also Figure S3). The light green line represents the measured signal intensity ratio, and the dotted line represents the standard deviation over the measurement time from 0 to 30 s. Data are plotted as the difference versus the mean for each laser diameter. Basically, as the values of both parameters increase, the amount of sample introduced increases and the SD decreases. Note that only panel A has a wider range of units displayed.

The laser repetition frequency and laser diameter were changed to examine the effect of each measurement on the magnitude of the standard deviation of the signal intensity ratio of Mg and Sr to Ca (Figure 2). The overall trend is for measurement stability to improve with increasing frequency and laser diameter. For the smallest laser diameter of 10 μm, the standard deviation increased by factors 2 to 3 compared to the larger diameter analysis (Figure S3). Under the conditions of 30, 50, and 70 μmφ (Figure 2B–D), the SD values were stable at frequencies above 10 Hz.

**FIGURE 3:**
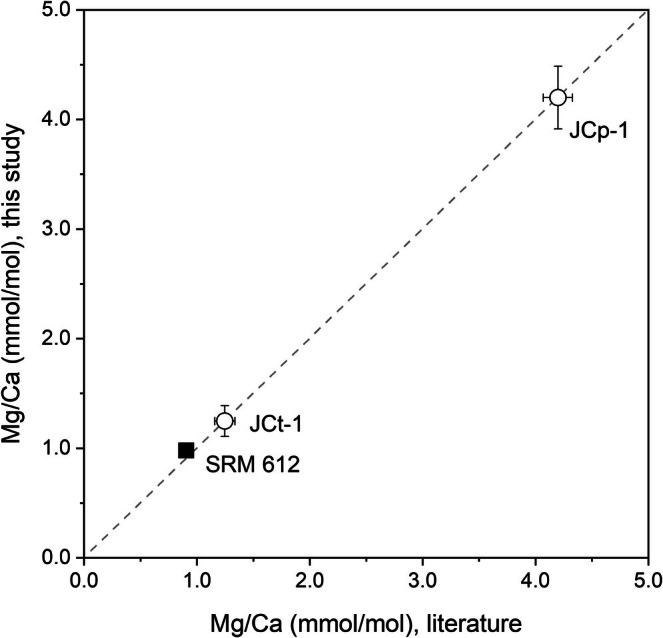
Comparison of fsLA‐ICP‐MS Mg/Ca data (mmol/mol) for carbonate (white circles) and glass (solid square) reference materials. Reference values were taken from the GeoReM database for NIST SRM 612 and Hathorne et al. [60] for JCp‐1 and JCt‐1.

The Mg/Ca data for the carbonate reference material and the glass reference material were also quantified based on the element ratios of the carbonate standard during the same analytical session, confirming a 1:1 relationship with the literature values, respectively (Figure 3). No element partitioning due to material differences was observed.

### 3.2 Verification of Cleaning Methods Using Different Alkaline Reagents

We prepared three replicate samples, each containing approximately 4 mg of two particle size fractions of 63–150 μm and 150–250 μm. Regarding the sediment sample characteristics, foraminifera and calcareous nannofossils are the most abundant components in these sediments. Microscopic analysis indicates that the abundance of foraminifera and calcareous nannofossils varies between 23%–56% and 36%–65% of the total sediment, respectively [63]. Clay minerals are also universally present, accounting for 7%–13% in smear preparations, and siliceous microfossils, such as radiolarians and sponges, are present in trace amounts and scattered throughout the entire core [63]. Most of the nannofossils and clay, which are fine‐grained constituents other than foraminifera, were removed by pre‐cleaning using ultrapure water and methanol. Following this, small siliceous materials were extracted under a microscope. This was followed by cleaning with reagents.

**FIGURE 4:**
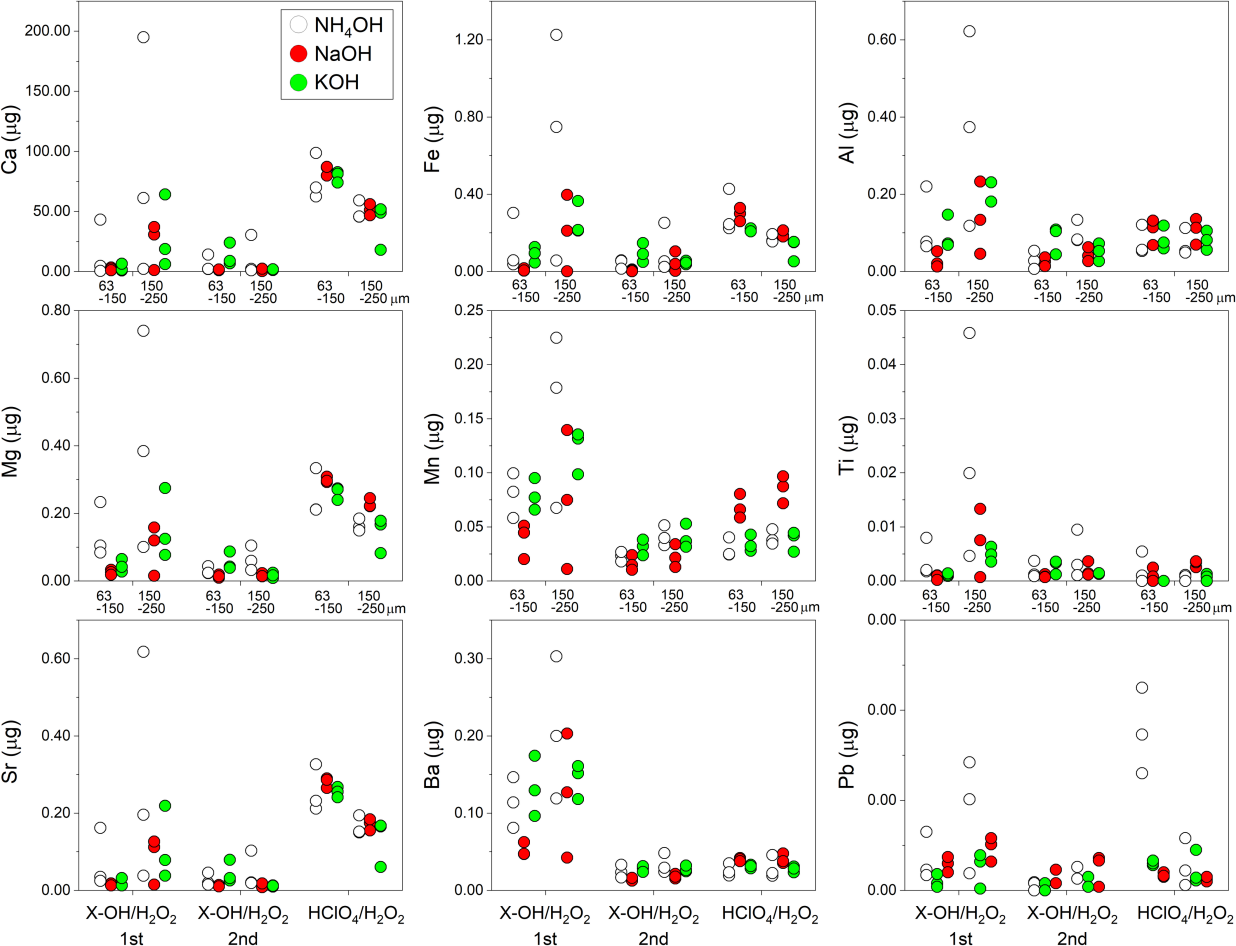
Element content in the supernatant obtained after cleaning sediment samples using alkaline solutions of NH_4_OH, NaOH, and KOH. Two size fractions (63–150 μm and 150–250 μm) of the same sediment sample used for laser analysis, with 4 mg of material per sample (Table S5), were subjected to each sequential cleaning experiment.

The dissolution behavior of Ca, Mg, Sr, Fe, Mn, Ba, Al, Ti, and Pb contained in the supernatant of a mixture of X‐OH (where X denotes NH_4_, Na, or K)/H_2_O_2_ is shown in Figure 4. Sequential cleaning progresses from left to right across each panel in the figure. Overall, the X‐OH/H_2_O_2_ shows high contents for most elements during the first cleaning step, with some samples exhibiting spikes in concentration. During the second X‐OH/H_2_O_2_ cleaning, the overall elution amount decreases. These behaviors are thought to originate from fine carbonate impurities, such as nanofossils, and clay minerals present on the surface of foraminifera tests and between particles.

**FIGURE 5:**
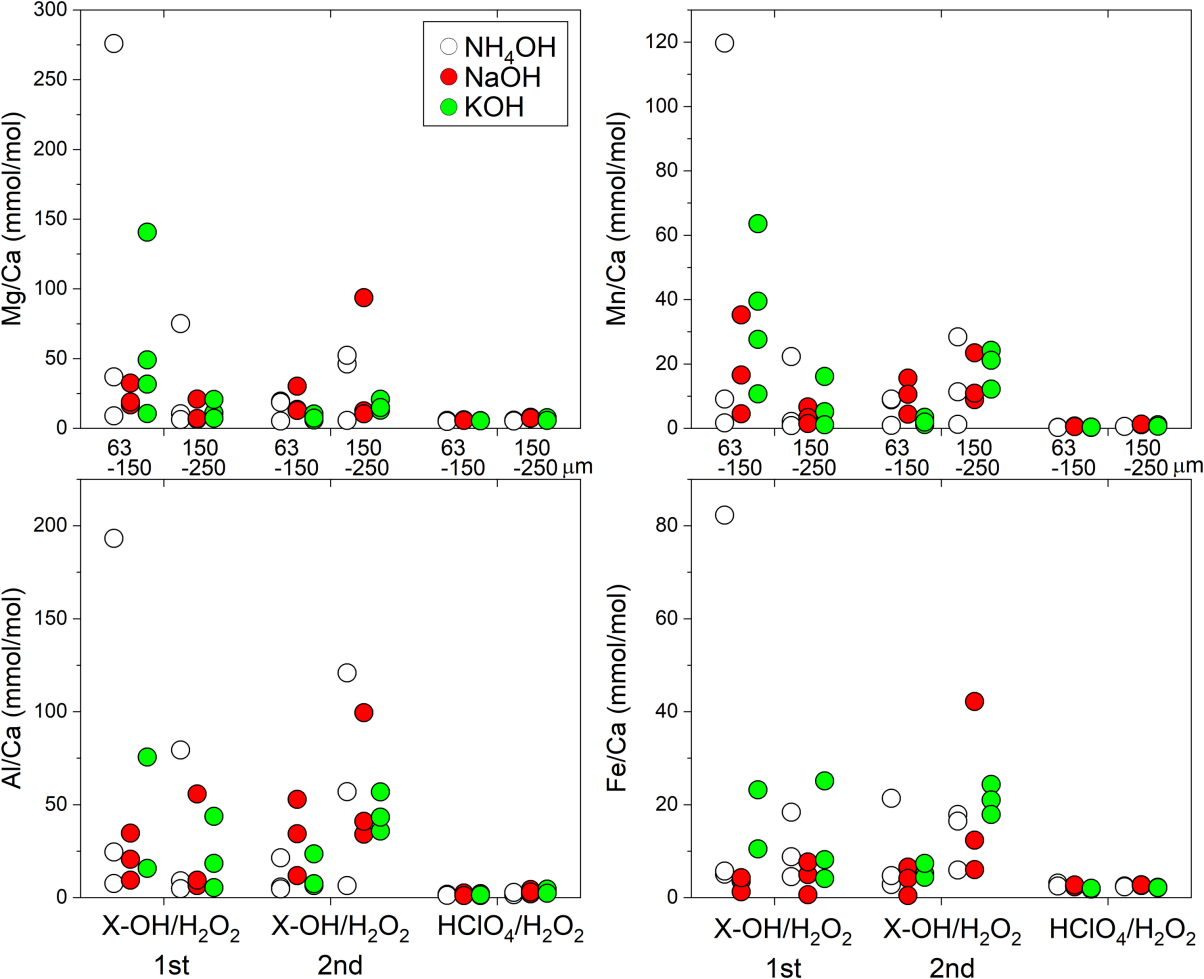
The Ca ratio of elements in the supernatant of Figure 4, showing a decrease of up to two orders of magnitude as the cleaning procedure progresses from left to right of each panel. The Mg/Ca and Sr/Ca ratios of cleaned foraminifera residues are listed in Table S6.

In the second cleaning solution, the HClO_4_/H_2_O_2_ mixture, Ca, Mg, and Sr exhibit relatively high concentrations. This synchronized behavior is thought to result from the partial dissolution of carbonate material due to the surface leaching. Supporting this, the Mg/Ca ratio shown in the figure below achieves values close to the composition of foraminifera. For smaller particle sizes (63–150 μm), Ca elution is higher, likely reflecting the larger surface area in contact with the solution due to size dependence. The measured Mg/Ca ratio, along with Al/Ca, Mn/Ca, and Fe/Ca indicating impurity contributions, showed highly variable and elevated values in the X‐OH/H_2_O_2_ cleaning solution (Figure 5). However, in HClO_4_/H_2_O_2_, these values were up to one or two orders of magnitude lower, clearly indicating that most impurities were effectively removed. Other elements either exhibited behavior similar to these four ratios or showed smaller changes.

**FIGURE 6:**
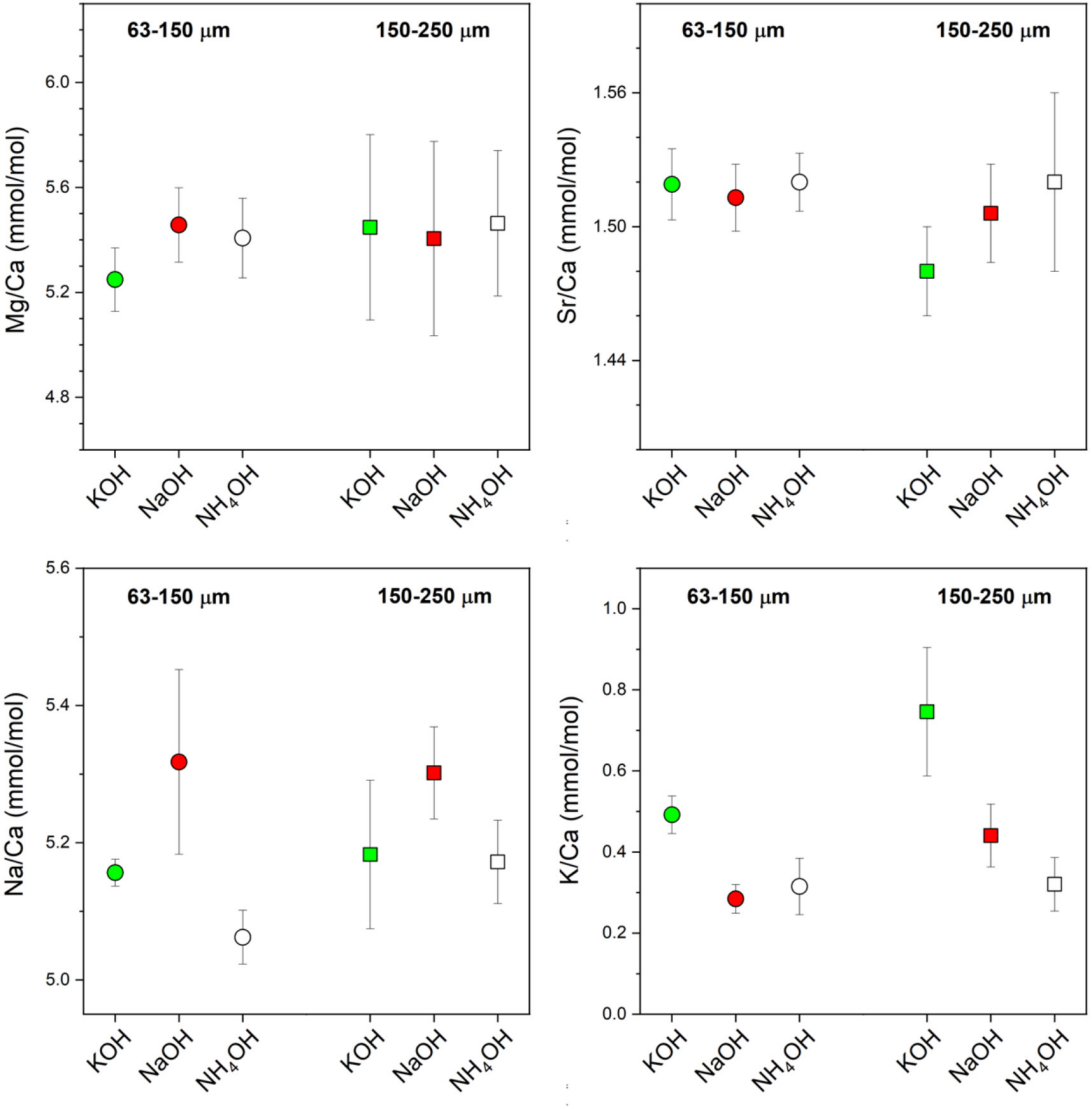
Ratios of Mg/Ca, Sr/Ca, Na/Ca, and K/Ca in residual foraminifera tests obtained after oxidation cleaning. Two grain size fractions (63–150 μm and 150–250 μm) were extracted from the same sediment sample used for laser analysis. For each sample, 4 mg of foraminifera tests were subjected to sequential cleaning without species sorting. Error bars indicate 1SD values for three replicates.

Finally, Figure 6 presents the element compositions of foraminiferal tests and the residues remaining after cleaning with each reagent. Potential carryover from the reagents was of concern for Na/Ca in the NaOH method and for K/Ca in the KOH method. We also evaluated whether each reagent could induce measurable offsets in Mg/Ca and Sr/Ca. Analyses of cleaned tests from the two size fractions (63–150 μm and 150–250 μm) indicated that the average values calculated for three specimens increased for Na/Ca following NaOH cleaning and for K/Ca following KOH cleaning. The Na/Ca increase was relatively small (2%–4%), whereas potential contamination of K/Ca was substantial (approximately 64%–96%). This memory effect was therefore considerably stronger for potassium, although no influence was detected for the other measured parameters.

Notably, no differences in Mg/Ca or Sr/Ca—key paleoceanographic proxies—were observed among the three reagents (NH_4_OH, NaOH, and KOH). Carryover from NaOH and KOH was also evident in the H_2_O_2_–HClO_4_ cleaning solution (Figure S4). Nonetheless, Mg/Ca and Sr/Ca ratios tended to converge toward values characteristic of dissolved and more purified test material. Accordingly, while the choice of cleaning reagent exerts minimal influence on Mg/Ca and Sr/Ca, NH_4_OH cleaning is recommended when simultaneously measuring Na/Ca and K/Ca, which have recently gained attention as geochemical proxies. Therefore, equivalent cleaning effects can be achieved with any reagent, allowing selection based on the target measurement, such as Na/Ca, K/Ca, or δ^41^K [45, 64].

### 3.3 Reproducibility and Different Cleaning Protocols for Foraminifera TE/Ca

The calibration curves for TE/Ca analysis of foraminifera tests were created after repeated analysis of the standards to cover the signal intensity range of the sample. The different Mg/Ca values of JCp‐1 and JCt‐1 were compared with their international consensus Mg/Ca values [60], and calibration curves were generated for each element using JCp‐1 and JCt‐1 (Figure 1).

The spherical morphology of *O. universa* has been reported to be suitable for confirming the reproducibility of TE/Ca composition and laser measurement conditions since element distribution in the depth direction of the test has been reported to show high reproducibility [34, 35]. Multiple individuals of *O. universa* were obtained from 0 to 5 cm and 5 to 8.5 cm stratigraphic levels, each of which is considered to suffer from the same degree of contamination in the sediment. Two different cleaning methods were therefore applied (see Section 2.3) to test whether differences in the reproducibility of the analyzed values arise when the same chamber is measured multiple times. The results of repeated measurements of *O. universa* and *P. obliquiloculata* are shown in Table 1. Mg/Ca ratios of H_2_O_2_–HClO_4_ cleaned final spherical chamber of *O. universa* showed excellent homogeneity with relative standard deviation (RSD) values within 3.7%–6.1%.

The Mg/Ca values seen in different individuals of *O. universa* cleaned using the H_2_O_2_–HClO_4_ method range from 4.73 to 8.90 mmol/mol (Table 1). Ultrasonic UPW‐MeOH cleaning resulted in 2–3 times larger errors of 13.0% and 8.3% for the Mg/Ca of two individual samples of *O. universa* at 0.0–5.0 cm. Oxidative cleaning yielded mean relative standard deviations of 3.9%, 3.4%, and 10.0% for Mg/Ca, Sr/Ca, and Na/Ca, respectively.

Averaging the RSD values for Mg/Ca of all UPW‐MeOH cleaned *O. universa* and *P. obliquiloculata* individuals produced about twice as much heterogeneity (7.5%) compared to oxidative cleaning (3.9%). Sr/Ca analysis is independent of cleaning and degrees of contaminant amounts (3.2% vs. 3.4% for UPW‐MeOH and H_2_O_2_–HClO_4_ cleaning, respectively), since similar SD values between the cleaning protocols were observed. Na/Ca also showed no systematic increase in RSD, but included several individuals with Na/Ca > 10 mmol/mol regardless of the cleaning method. This large variation may reflect actual differences at the individual level, but individual analysis shows far greater variability than bulk mixed‐species analysis (Figure 6). It is also possible that localized internal contamination may occur within the test or elsewhere.

### 3.4 Chamber‐Specific Analysis

**FIGURE 7:**
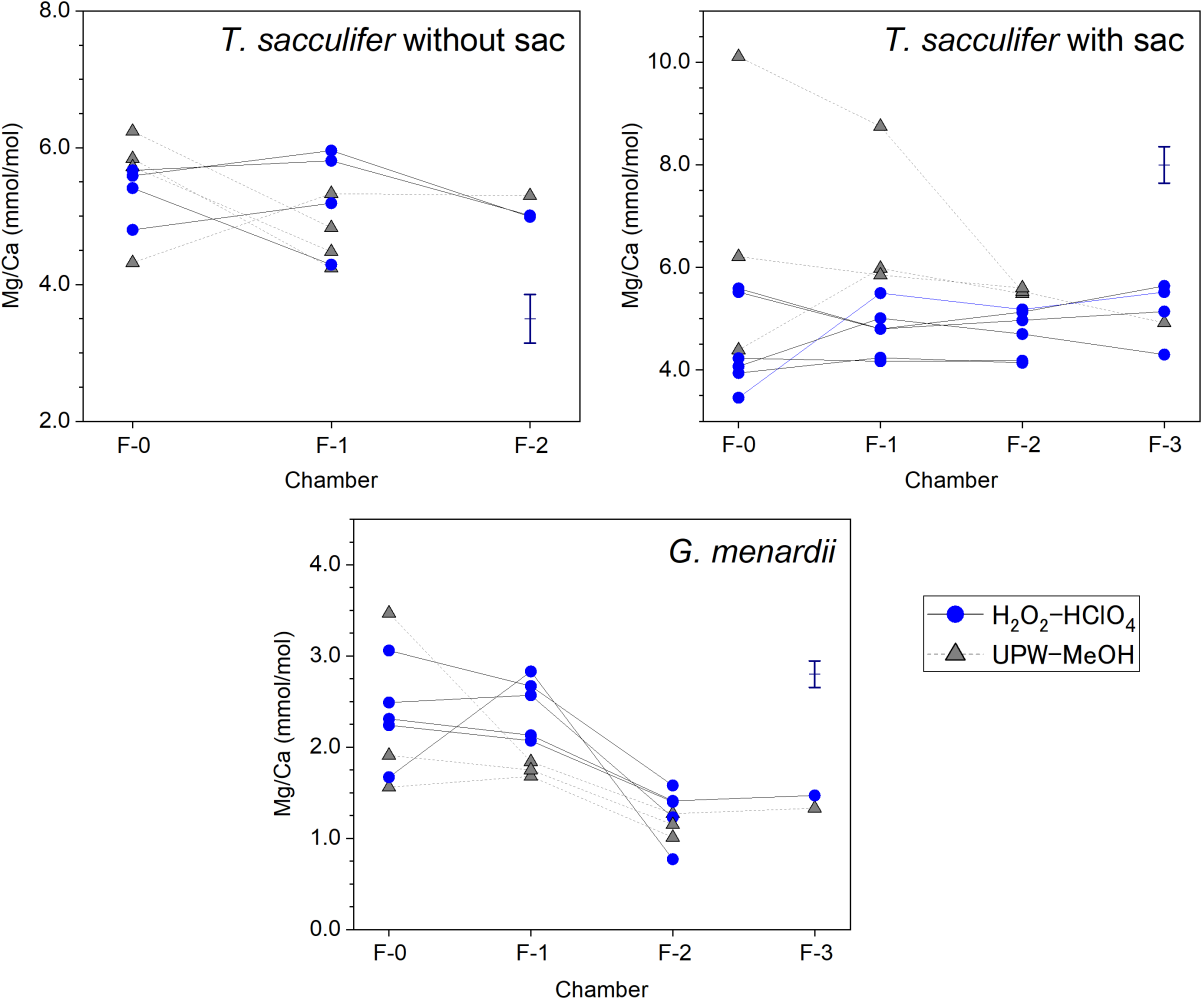
Chamber‐specific Mg/Ca for each individual of *T. sacculifer* with sac, without sac, and *G. menardii*. Triangles for cleaning with ultrapure water and methanol (UPW‐MeOH), and circles with hydrogen peroxide and perchloric acid (H_2_O_2_‐HClO_4_). The navy blue error bars of each panel indicate the 2SD reproducibility values.

Chamber‐specific Mg/Ca, Sr/Ca, and Na/Ca results for *T. sacculifer* and *G. menardii* are shown in Tables 2 and S1–S3. Vertically resolved sampling of four species of planktonic foraminifera from the tropical Atlantic showed that more than half of the individuals collected during the survey do not follow a trajectory synchronous with species‐typical ontogenetic vertical migration [53]. It is also well known that at the individual level there is variation between individuals reflecting differences in the seasons inhabited and life history, e.g., [13, 16]. Given the sediment sample assemblages probably contain a mix of specimens recording a variety of individual trajectories in time and space, TE/Ca are discussed considering the average and statistical variance for each individual chamber (Figure 7, see also Figure S5). We determined the average value for each chamber. *T. sacculifer* without sac could be analyzed from F0 to F2, and *T. sacculifer* with sac could be analyzed from F0 to F3. For H_2_O_2_–HClO_4_ cleaned samples, each *T. sacculifer* (with and without sac) showed similar Mg/Ca ratios among the chambers. There is a difference in the effects of pre‐cleaning differed between the two morphological types, which may be related to the thinness of the test. In some UPW‐MeOH cleaned *T. sacculifer* with sac, the last two chambers (F0 and F1) showed very high Mg/Ca and Na/Ca values. On the other hand, in *T. sacculifer* without sac, the differences between cleaning methods were small.

For *G. menardii*, Mg/Ca tended to increase with chamber addition (Figure 7, Table 2); rather than a gradual increase from F3 to F0, it was around 1.4 mmol/mol in F2 and F3 but 2.5 mmol/mol in F1 and F0. Compared with *T. sacculifer*, *G. menardii* showed less difference between cleaning methods.

**TABLE 2:** Mean and standard deviation of element ratios calculated for each chamber of *T. sacculifer* and *G. menardii*. Results are shown separately for cleaning with ultrapure water and methanol (UPW‐MeOH), hydrogen peroxide and perchloric acid (H_2_O_2_–ClO_4_).

	*N*	Mg/Ca	SD	Sr/Ca	SD	Na/Ca	SD

mmol/mol		mmol/mol		mmol/mol	
*T. sacculifer* without sac, H_2_O_2_–HClO_4_							
F0	4	5.37	0.40	1.46	0.08	8.30	0.87
F1	4	5.31	0.76	1.43	0.11	8.84	1.81
F2	2	5.00	0.01	1.36	0.07	8.76	2.45
*T. sacculifer* without sac, UPW‐MeOH							
F0	4	5.53	0.84	1.50	0.03	7.91	1.15
F1	4	4.72	0.47	1.41	0.10	6.89	1.21
F2	1	5.30	—	1.65	—	7.37	—
*T. sacculifer* with sac, H_2_O_2_–HClO_4_							
F0	6	4.47	0.88	1.47	0.09	7.53	2.84
F1	6	4.75	0.50	1.39	0.04	5.41	1.37
F2	6	4.72	0.46	1.40	0.05	4.72	1.10
F3	4	5.15	0.60	1.40	0.04	5.22	1.38
*T. sacculifer* with sac, UPW‐MeOH							
F0	3	6.91	2.92	1.39	0.09	21.82	16.48
F1	3	6.86	1.64	1.40	0.10	11.56	9.02
F2	3	5.54	0.06	1.40	0.09	6.26	1.40
F3	1	4.92	—	1.38	—	6.39	—
*G. menardii*, H_2_O_2_–HClO_4_							
F0	5	2.35	0.50	1.44	0.07	7.22	0.88
F1	5	2.45	0.34	1.42	0.07	6.56	1.01
F2	5	1.28	0.31	1.25	0.10	5.30	0.31
F3	1	1.47	—	1.30	—	5.10	—
*G. menardii*, UPW‐MeOH							
F0	4	2.57	0.98	1.56	0.34	6.03	0.57
F1	4	2.23	0.94	1.33	0.09	5.55	0.84
F2	4	1.31	0.35	1.30	0.12	5.05	0.29
F3	1	1.33	—	1.21	—	5.03	—

### 3.5 Optimization of Analytical Protocols

In our fsLA‐ICP‐MS system, the element ratios were uniform regardless of changes in Ca intensity (Figure 1), but the stability of the respective measurements increased with increasing laser spot size (Figure 2). Therefore, in order to increase the signal intensity as much as possible, a spot size of 30 μm or more was set, which is a maximum for foraminifera chamber analysis. Because the time required to penetrate the foraminifera test becomes shorter as the repetition frequency is increased, we used 5–15 Hz to measure the foraminiferal chamber. The timing of the penetration was determined by the tendency of the signal intensity of elements: decreasing timing for Ca, whereas increasing for Mg, as well as Al and Ba signals (data not shown), which are the elements commonly attaching fine particles inside the test.

As for test cleaning, the reproducibility of Mg/Ca was improved by oxidative cleaning, while those of Sr/Ca and Na/Ca were not so different between the two cleaning processes. Considering that the oxidative treatment more effectively removes contamination sources compared to the UPW‐MeOH cleaning, very fine sediments or precipitates such as Mn (–Fe) oxyhydroxide could potentially be the cause. Since the ionic radius of Mn^2+^ is closer to that of Mg^2+^ than that of Sr^2+^ or Na^+^, Mg is more likely than Sr or Na to substitute for Mn in Mn oxyhydroxides.

## 4 Application

### 4.1 Habitat Temperature Reconstruction

The habitat temperature at which foraminifera lived was reconstructed for each chamber based on the Mg/Ca data obtained by fsLA‐ICP‐MS (please refer to Table 3 and the Supporting Information for the equation used). For *T. sacculifer*, reconstructions of habitat temperature were attempted only for H_2_O_2_–HClO_4_ cleaned individuals with sac and without sac. The average habitat temperature of all measured *T. sacculifer* individuals was calculated to be 29.5 ± 0.6°C (see Supporting Information for Mg/Ca temperature equations). This is consistent with the mean water temperature of 29.2°C in the mixed layer at a depth of 0–50 m calculated from the temperature profile of the WOA near the sample collection site (Figure S1). This means that the measurement accuracy of the fsLA‐ICP‐MS is guaranteed from the perspective of comparison with environmental data.

**TABLE 3:** A list of representative temperature conversion equations for the four species of foraminifera measured in this study and the mixed species that included them [5, 7, 43, 48, 49, 65, 66, 67].

Species	Locality	Type	Mg/Ca = B exp. (AT)				References
			B	±	A	±	
8 species^a^	N. Atlantic	Core top	0.65	0.04	0.085	0.005	Elderfield and Gannsen, [5]
10 species^b^	N. Atlantic	Sediment Trap	0.38	0.02	0.090	0.003	Anand et al. [66]
7 species^c^	Eq. Pacific	Core	0.455		0.077		Sagawa et al. [48]
Shallow‐thermocline 4 species^d^	Eq. Atlantic	Core	0.22		0.113		Regenberg et al. [67]
Shallow‐thermocline 4 species	Eq. Atlantic	Core	0.29		0.101		Regenberg et al. [67]
*T. sacculifer*, *N. pachyderma*		Culture, core top	0.47	0.03	0.082	0.006	Nürnberg et al. [17]^e^
*T. sacculifer*		Culture	0.39	0.06	0.089	0.008	Nürnberg et al. [17]^e^
*T. sacculifer*	Global	Core top	0.37		0.090		Dekens et al. [49]^f^
*T. sacculifer* with sac	N. Atlantic	Sediment trap	0.67	0.31	0.069	0.013	Anand et al. [66]
*T. sacculifer* without sac	N. Atlantic	Sediment trap	1.06	0.21	0.048	0.012	Anand et al. [66]
*T. sacculifer* with sac	N. Atlantic	Sediment trap	0.377	0.010	0.090		Anand et al. [66]^g^
*T. sacculifer* without sac	N. Atlantic	Sediment trap	0.343	0.011	0.090		Anand et al. [66]^g^
*T. sacculifer*			0.60	0.16	0.075	0.006	Regenberg et al. [67]
*G. menardii*	Eq. Atlantic	Sediment surface	0.36	0.31	0.091	0.012	Regenberg et al. [67]
*G. menardii*	South China Sea	Sediment surface	0.53	0.11	0.069	0.005	Regenberg et al. [65]
*O. universa*		Culture	1.36	0.24	0.085	0.011	Lea et al. [43]
*O. universa*	N. Atlantic	Sediment trap	0.595	0.042	0.090		Anand et al. [66]^g^
*P. obliquiloculata*	N. Atlantic	Sediment trap	0.18	0.10	0.120	0.030	Anand et al. [66]
*P. obliquiloculata*	N. Atlantic	Sediment trap	0.532	0.008	0.090		Anand et al. [66]^g^

^a^ *G. bulloides* , *G. ruber* , *G. sacculifer*, *G. siphonifera* , *N. pachyderma*, *G. hirsuta* , *G. inflata* , *G. truncatulinoides*.

^b^ *G. ruber* white/pink, *G. sacculifer*, *G. conglobatus* , *. aequilateralis* , *O. universa* , *N. dutertrei*, *P. obliquiloculata, G. inflata, G. truncatulinoides, G. hirsuta, G. crassaformis*.

^c^ *G. ruber* , *G. sacculifer*, *G. aequilateralis* , *G. conglobatus* , *P. obliquiloculata*, *G. menardii*, *G. tumida*.

^d^ *G. ruber* , *G. sacculifer*, *G. menardii*, *N. deutertrei*.

^e^ Values from Anand et al. (2003).

^f^ A dissolution correction based on core depth has also been proposed.

^g^ A temperature dependence was assumed to be the same as multi‐species calibration.

**FIGURE 8:**
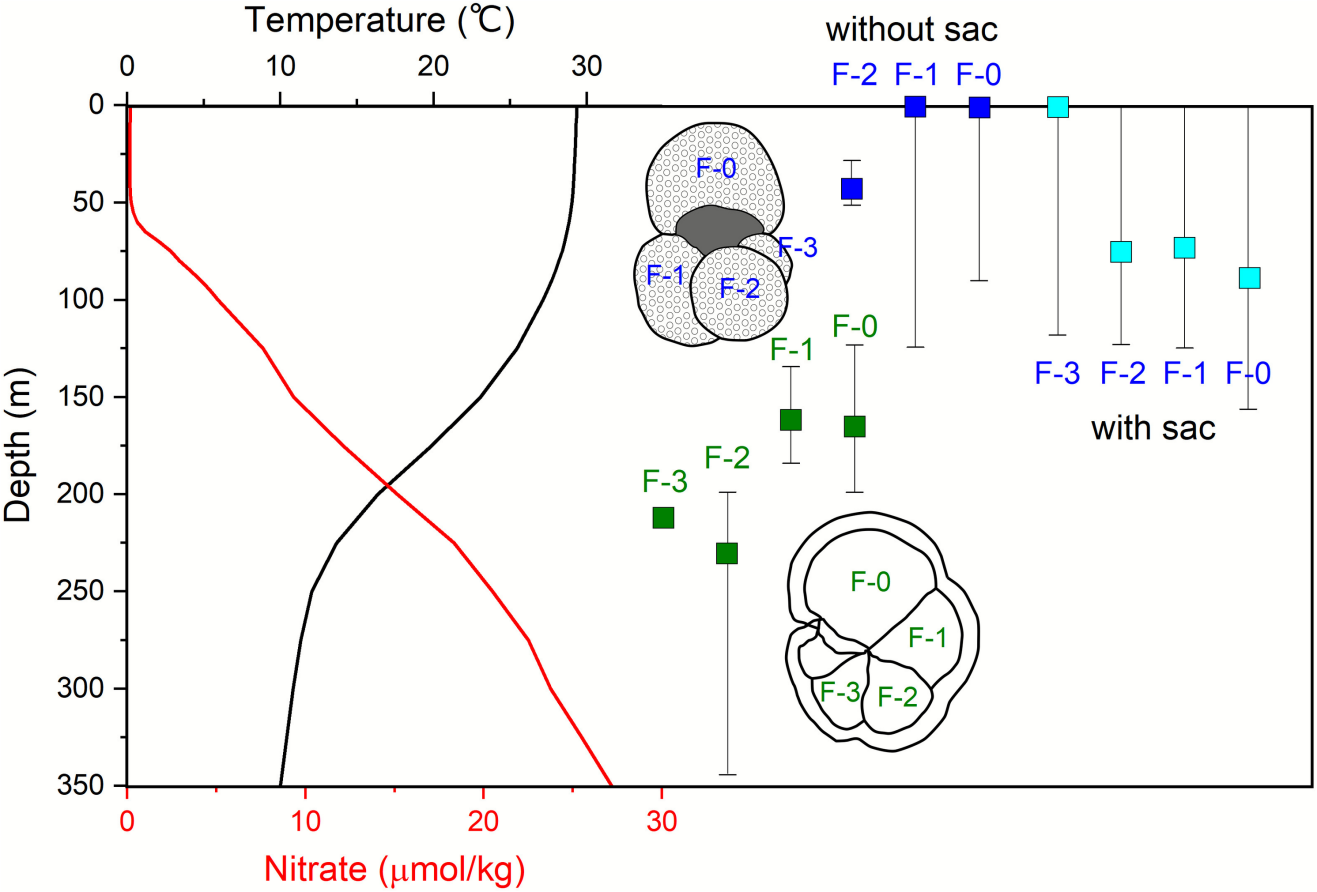
Chamber‐specific Mg/Ca temperature in H_2_O_2_–HClO_4_ cleaned *T. sacculifer* and *G. menardii* converted to water depth in modern time (see Figure S1). The water temperature conversion equations for *T. sacculifer* and *G. menardii* were average of each species‐specific calibration curves (Table 3). Error bars indicate the variation in calcification depth corresponding to the measurement errors of Mg/Ca in Table 2.

The chamber‐specific temperature reconstructions for *T. sacculifer* without sac were 29.9 ± 2.2°C (*n* = 4) at F0, 29.8 ± 4.3°C (*n* = 4) at F1, and 29.1 ± 0.1°C (*n* = 2) at F2, consistent within a 1SD range, indicating that they lived only inside the mixed layer (Figure 8). The chambers of *T. sacculifer* with sac agree with each other: 27.8 ± 5.5°C (*n* = 6) at F0, 28.5 ± 3.0°C (*n* = 6) at F1, 28.4 ± 2.8°C (*n* = 6) at F2, and 29.4 ± 3.5°C (*n* = 4) at F3, which are slightly lower than that of *T. sacculifer* without sac (Figure 8). The test of *T. sacculifer* contains gametogenic calcite, and the formation of the sac‐like final chamber precedes gametogenesis by about 24–48 h [51]. Sedimentary foraminiferal tests always contain a mixture of gametogenic and pre‐gametogenic calcite, resulting in potential heterogeneity in Mg/Ca in *T. sacculifer* and other species with high gametogenic calcite abundance.

Statistical analysis for this difference in Mg/Ca per chamber with and without sac was performed, and significant differences could be detected only in the following two conditions (Supporting Information): (1) between H_2_O_2_–HClO_4_ and UPW‐MeOH treatments and (2) between H_2_O_2_–HClO_4_ cleaned *T. sacculifer* with sac and without sac (Table S4). For the former, the results support the effectiveness of oxidation cleaning. The latter results indicate that the difference of calcification temperature between *T. sacculifer* without and with sac is 1.4°C, with the former calcifying at shallower than 40 m, while the latter calcifies at about 80 m. Our result confirms that the without‐sac morphology is a suitable paleoceanographic tracer that provides more accurate temperature records for the surface mixed layer.

Mg/Ca ratio of *G. menardii* increased toward the final chamber in most individuals, suggesting their depth migration associated with growth. For *G. menardii*, thermocline development and food availability have been proposed as factors in its distribution [1]. The initiation of globorotaliids calcification occurs in relatively warm waters above the seasonal thermocline of preferred food availability [1, 50, 52]. In plankton nets collected in April in the eastern equatorial Pacific, *G. menardii* shows two frequent depth occurrences, one at 30–34 m just below the mixed layer and the other at the deep chlorophyll maximum of 52–63 m [1]. Final chamber formation of *G. menardii* in the South China Sea is estimated to occur at about 200 m (about 15°C) near the bottom of the seasonal thermocline [65].

Calculated calcification temperatures were 21.1 ± 4.5°C (*n* = 5) for F0, 21.6 ± 3.0°C (*n* = 5) for F1, 13.4 ± 3.2°C (*n* = 5) for F2, and 15.1°C (*n* = 1) for F3 (Figure 8). Interestingly, the calcification temperature was found to indicate shallower depths with chamber accretion. Converting this temperature to depth based on the WOA data revealed that from F3 to F0, the calculated calcification depth shifted from > 200 m at the deepest depth to ~160 m at the top of the thermocline. Although this is an individual‐level analysis, the value obtained by picking 62.1 mg of bulk *G. menardii* tests from the same surface sediment (meaning the same batch) by ICP‐MS is 2.37 mmol/mol and the converted water temperature is 21.2°C [64]. This result means that the bulk analysis of *G. menardii* is weighted by the heavy F0 and F1 and retains the paleoceanographic record of the upper part of the thermocline in the WPWP. In the South China Sea, the average Mg/Ca of the bulk test of *G. menardii* was about 2.17 mmol/mol, corresponding to a calcification temperature of 20.2 ± 1.9°C [65]. Based on paired oxygen isotope and Mg/Ca analysis, *G. menardii* from surface sediments of the South China Sea is a seasonal thermocline resident [65]. Although this species is known to be ecologically sensitive to nutrients, the good agreement in both regions in terms of calcification temperature helps to constrain habitat conditions.

*O. universa* is known to exhibit systematically higher Mg/Ca values than other species at the same habitat temperature, and the data variability is also known to be high in sediment traps and in cultured samples [66]. Mg/Ca temperature ranged from 14.7°C to 22.1°C using the equation of Lea et al. [43] and from 23.0 to 30.1°C using Anand et al. [66], suggesting that there were > 7°C differences in calcification temperature of spherical final chamber among individuals. *O. universa* is known to be collected over a very wide range from the surface mixed layer to the thermocline in modern samples, and core‐top samples also show a large variation in calcification temperature [68]. Therefore, it is supported that the spherical chambers do not calcify in a certain depth range for the present sample as well. In the UPW‐MeOH‐cleaned samples, no systematic difference was observed for Sr/Ca, but only Mg/Ca tended to be higher (up to 12.55 mmol/mol), indicating that they are susceptible to contamination.

Results of a plankton net survey on the Manihiki Plateau indicate that all samples of *P. obliquiloculata* are calcified between 145 and 170 m, which is suitable for thermocline reconstruction [4]. The calcification depth converted using Sagawa's equation is 115–175 m [42]. Similar to that reported by *G. menardii*, Li et al. [57] have also reported Mg/Ca of this species using 62.4 mg of bulk tests as 3.09 mmol/mol, which corresponds to a calcification temperature of 24.9°C and a water depth of 131 m, as estimated from Sagawa's formula [48]. Although individual analysis by LA‐ICP‐MS suggested a deeper than the depth estimated from bulk analysis, the large bulk analysis is in good agreement with the upper thermocline of the vertical thermal structure in WPWP.

### 4.2 Na and Sr Partitioning

In addition to Mg/Ca thermometers, uptake of other trace elements has been calibrated as proxies in paleoceanography. Sr/Ca ratios in foraminiferal tests have been reported to increase with calcification rate [7, 43]. This response is particularly pronounced in response to ambient dissolved inorganic carbon (DIC) concentrations (Allen et al. [10] and further references therein). Carbonate‐system parameters are closely linked to foraminifera calcification, and their influence on Sr incorporation into calcite is supported by both experimental and theoretical studies, e.g., [69, 70]. Higher calcite precipitation rates are associated with increased Sr partition coefficients, and the influence of seawater carbonate chemistry is also evident in foraminiferal Mg/Ca, Sr/Ca, and Mn/Ca ratios [43, 71, 72]. However, the fine‐scale variation in Sr/Ca within tests is relatively small, in contrast to the pronounced diurnal Mg/Ca variations [72].

The Sr/Ca ratio in the foraminifera tests of many planktonic foraminifera is relatively unaffected by temperature or salinity, e.g., [10, 43]. Our chamber‐specific analysis also shows a very homogeneous Sr distribution (Table 2). These results would mean that there is no competition between Sr and Mg partitioning, i.e., ambient temperature. The homogeneity of Sr/Ca ratios at the chamber level may reflect the small range of variation in oceanic carbonate system parameters in the study area.

The residence time of sodium in seawater is long, approximately > 50 million years [73, 74, 75], suggesting that its concentration is expected to remain relatively stable in the short term. In contrast, the residence time for calcium is two orders of magnitude shorter, at 1 million years. Consequently, the Na/Ca ratio in foraminifera has been proposed as a direct proxy for seawater calcium concentration [11, 45]. Alternatively, it is a proxy dependent on seawater salinity and Na/Ca ratios [9].

To date, influences on Na/Ca have been proposed including Mg/Ca fluctuations, salinity, diagenetic changes, individual variation, chamber‐specific differences, and interspecific variation [9, 10, 11, 33]. The amount of specific Na concentrated parts in the test, represented by spines [9], increases with extremely high salinity or Na^+^/Ca^2+^ activity ratios. In addition, Gray et al. [76] showed that there are systematic Na/Ca offsets between live‐collected and core top foraminifera, that is best explained by a readily‐lost Na‐bearing phase. If these primary phases remain, they will result in high Na/Ca. However, when dealing with ordinary open ocean settings, salinity variations are expected to be negligible and therefore other factors are more likely to control variable Na/Ca in foraminiferal tests. Our individual‐level analysis detected uniquely high individual variation in Na/Ca ratios in *O. universa* and other species, and this variation does not necessarily show a consistent trend with Mg/Ca. While the use of proxies always emphasizes the importance of species‐level selection, selecting more stable Na uptake conditions at the chamber level would likely be suitable for reconstructing geological‐era Ca concentrations.

Calcium carbonate is not only found in the hard parts of organisms, which are environmental recorders from the geological era, but also in various Earth's rocks (e.g., REE deposits [77]) and even in asteroid samples [78], making it an excellent recording material for aqueous chemistry, physical, and ecological conditions. The methodology for micro‐sampling using a femtosecond laser established in this experiment makes it possible to obtain highly accurate element ratio data from carbonates.

## 5 Conclusion

Femtosecond LA‐ICP‐MS analysis was used to report on changes in trace element to Ca ratios of within and between individual foraminifera CaCO_3_ tests. For fsLA‐ICP‐MS analysis, a spot size of 30 μm or larger was used at a laser repetition frequency of 5–15 Hz in a circular analytical trajectory, and stable signal intensities for Ca and trace elements were obtained. Quantitative analysis was accomplished with carbonate standard materials, showing good linearity with the NIST SRM 612 glass. Differences between pre‐cleaning methods, primarily for particulate removal (distilled water + methanol) and chemical oxidation treatment (perchloric acid and hydrogen peroxide), were also examined. The use of an oxidative cleaning treatment improved reproducibility. *T. sacculifer* without sac calcified in the mixed layer of the marine water column and is suitable as a proxy of sea surface temperature. The Mg/Ca water temperature of *T. sacculifer* with sac was about 1.4°C lower than the *T. sacculifer* without‐sac and precipitated their final calcite layer at slightly deeper depths. *G. menardii* showed a migration of 6°C–8°C to the higher temperature from F3 and F2 to the final chamber in all samples. Records of the upper part of the thermocline were obtained from the final F0 and F1 chambers of *G. menardii*. Na/Ca is nearly constant in *T. sacculifer* and seems to have a unique apparent partition coefficient in species that inhabited mixed layers with constant water temperature. Differences in Sr/Ca between chambers and between species were also small.

## Author Contributions

**Toshihiro Yoshimura:** conceptualization, funding acquisition, investigation, resources, methodology, writing – original draft, visualization, and writing – review and editing. **Qing Chang:** conceptualization, funding acquisition, investigation, methodology, writing – original draft, visualization, and writing – review and editing. **Naohiko Ohkouchi:** resources, visualization, and writing – review and editing. **Yoshiyuki Ishitani:** conceptualization, funding acquisition, investigation, formal analysis, and writing – review and editing. **Dana Ulanova:** conceptualization, funding acquisition, investigation, and writing – review and editing. **Hirotoshi Endo:** conceptualization, funding acquisition, investigation, and writing – review and editing. **Junichiro Kuroda:** resources and writing – review and editing. **Yurika Ujiié:** conceptualization, funding acquisition, investigation, methodology, writing – original draft, and writing – review and editing.

## Funding

This work was performed with the support of Japan Society for the Promotion of Science (JSPS) to Y.U., Y.I., D.U., E.H., and T.Y. (no. 20H02016 and 23H01290), TY (no. 21H01204), and T.Y. and J.K. (no. 21H01203).

## Conflicts of Interest

The authors declare no conflicts of interest.

## Supporting information


**Figure S1:** Vertical profiles of water temperature, salinity, and nutrient concentrations (nitrate and phosphate) at the WOA site near the sample collection site.
**Figure S2:** Examples of laser‐analyzed individual samples. The laser sampling spots of *O. universa* (right) and *P. obliquiloculata* (left) used in the repeated measurements are generally around 40–60 μm in size.
**Figure S3:** Experiments in LA‐ICP‐MS analysis with a fixed repetition rate of 10 Hz and laser sizes of 10, 30, and 50 μm. Three repetitions of each condition are shown for the JCp‐1 sample, and the average values are shown. Larger laser diameters provide stronger signal intensity and thus improve the signal‐to‐noise ratio of the measurements.
**Figure S4:**. Comparison of Mg/Ca, Sr/Ca, Na/Ca, and K/Ca ratios in residual foraminifera shells obtained after oxidation cleaning and in the second cleaning solution (H_2_O_2_‐HClO_4_). A dotted line at 1:1 indicates the effect of stoichiometric partial dissolution of the foraminifera shells for comparison. While Mg/Ca in most samples and Sr/Ca in nearly all samples are close to the composition of partially dissolved shells, Na/Ca and K/Ca show carryover of NaOH and KOH from the initial cleaning reagent.
**Figure S5:**. Two example of raw measurement data of *T. sacculifer* F1 (upper) and F0 chamber (lower) picked from 0 to 5 cm of the core. Each upper left panel shows the ratio of signal intensity for the target element to measurement time on the horizontal axis; lower left panel shows signal intensity versus time on the horizontal axis; upper right panel shows calcium signal intensity on the horizontal axis and signal intensity ratio on the vertical axis. Element ratios were determined during the calcium intensity plateau section. The section where signal intensity began to decrease indicates completion of the shell depth profile measurement and was not used for integration. In the region where calcium signal intensity is strong, stable element ratio signals can be obtained independently of the ^43^Ca signal intensity. The F1 shell is thicker than the F0 shell, resulting in a longer ablation time on the horizontal axis and yielding a smoothed depth profile via rotation raster mode.
**Table S1:** Data measured for each chamber in *G. menardii*.
**Table S2:** Data measured for each chamber in *T*. *sacculifer* with sac.
**Table S3:** Data measured for each chamber in *T*. *sacculifer* without sac.
**Table S4:** Results of statistical analysis examining differences in Mg/Ca in *T. sacculifer* by morphology and chamber.
**Table S5:** Element composition of cleaning supernatant. Two size fractions (63–150, 150–250 μm) of sediment samples from the Ontong Java Plateau were cleaned with reagents after removing fine‐grained material other than foraminifera using ultrapure water and methanol. The cleaning sequence was performed twice with a mixture of ammonium hydroxide, sodium hydroxide, potassium hydroxide, and hydrogen peroxide, followed by one treatment with a mixture of perchloric acid and hydrogen peroxide.
**Table S6:** Ratios of Mg/Ca, Sr/Ca, Na/Ca, and K/Ca in residual foraminifera shells obtained after oxidation cleaning. Two grain size fractions (63–150 μm and 150–250 μm) were extracted from the same sediment sample used for laser analysis. For each sample, 4 mg of foraminifera shells were subjected to sequential cleaning without species sorting.

## Data Availability

The datasets used and analyzed during the current study are available from the corresponding author on reasonable request.
