# Supplementary material for: Chamber‐by‐Chamber Measurements of Planktonic Foraminiferal Mg, Sr, and Na to Ca Ratios With Femtosecond LA‐ICP‐MS
Source: Rapid Commun Mass Spectrom. 2026 Jan 19;40(7):e70026. doi: 10.1002/rcm.70026 (PMC12816436; doi:10.1002/rcm.70026)
Supplement: Supplementary file 1 — Figure S1: Vertical profiles of water temperature, salinity, and nutrient concentrations (nitrate and phosphate) at the WOA site near the sample collection site. Figure S2: Examples of laser‐analyzed individual samples. The laser sampling spots of O. universa (right) and P. obliquiloculata (left) used in the repeated measurements are generally around 40–60 μm in size. Figure S3: Experiments in LA‐ICP‐MS analysis with a fixed repetition rate of 10 Hz and laser sizes of 10, 30, and 50 μm. Three repetitions of each condition are shown for the JCp‐1 sample, and the average values are shown. Larger laser diameters provide stronger signal intensity and thus improve the signal‐to‐noise ratio of the measurements. Figure S4:. Comparison of Mg/Ca, Sr/Ca, Na/Ca, and K/Ca ratios in residual foraminifera shells obtained after oxidation cleaning and in the second cleaning solution (H2O2‐HClO4). A dotted line at 1:1 indicates the effect of stoichiometric partial dissolution of the foraminifera shells for comparison. While Mg/Ca in most samples and Sr/Ca in nearly all samples are close to the composition of partially dissolved shells, Na/Ca and K/Ca show carryover of NaOH and KOH from the initial cleaning reagent. Figure S5:. Two example of raw measurement data of T. sacculifer F1 (upper) and F0 chamber (lower) picked from 0 to 5 cm of the core. Each upper left panel shows the ratio of signal intensity for the target element to measurement time on the horizontal axis; lower left panel shows signal intensity versus time on the horizontal axis; upper right panel shows calcium signal intensity on the horizontal axis and signal intensity ratio on the vertical axis. Element ratios were determined during the calcium intensity plateau section. The section where signal intensity began to decrease indicates completion of the shell depth profile measurement and was not used for integration. In the region where calcium signal intensity is strong, stable element ratio signals can be obtained i [file RCM-40-e70026-s001.docx]

Supporting Information

**Chamber-by-chamber measurements of planktonic foraminiferal Mg, Sr, and Na to Ca ratios with femtosecond LA-ICP-MS**

Toshihiro Yoshimura ^1*^, Qing Chang ^2^, Naohiko Ohkouchi ^1^, Yoshiyuki Ishitani ^3^, Dana Ulanova ^4, 5^, Hirotoshi Endo ^6^, Junichiro Kuroda ^7^, and Yurika Ujiié ^5^

^1^ Biogeochemistry Research Center, Japan Agency for Marine-Earth Science and Technology (JAMSTEC), Yokosuka, 237-0061, Japan

^2^ Volcanoes and Earth’s Interior Research Center, Japan Agency for Marine-Earth Science and Technology (JAMSTEC), Yokosuka, 237-0061, Japan

^3^ Institute for Extra-cutting-edge Science and Technology Avant-garde Research, Japan Agency for Marine-Earth Science and Technology (JAMSTEC), Yokosuka, 237-0061, Japan

^4^ Faculty of Agriculture and Marine Science, Kochi University, Monobe-Otsu 200, Nankoku, Kochi 783-8502, Japan

^5^ Marine Core Research Institute, Kochi University, Monobe-Otsu 200, Nankoku, Kochi 783-8502, Japan

^6^National Institute of Technology, Tsuruoka College, 104 Aza Sawada, Ioka, Tsuruoka, Yamagata 977-8511, Japan

^7^ Atmosphere and Ocean Research Institute, The University of Tokyo, 5-1-5 Kashiwanoha, Kashiwa, Chiba 277-0882, Japan

* Correspondence to T. Yoshimura, E-mail: [yoshimurat@jamstec.go.jp](mailto:yoshimurat@jamstec.go.jp)

**Supplementary Text**

***LA-ICP-MS***

The 200/266 femtosecond LA system uses an OK-Fs2000K (OK Laboratory, Tokyo, Japan), utilizing a Solstice one-box Ti:sapphire femtosecond regenerative amplifier (Spectra-Physics, Santa Clara, CA, USA) and TP-1A THG and TP-1A FHG frequency tripler and quadrupler harmonic generators (Spectra-Physics, Santa Clara, CA, USA).An excimer laser-grade high-power fused silica (266 nm) was used as the objective lens, with a laser fluence of 12 J cm^2^ at 266 nm on the sample surface. The rotating raster ablation mode employed to obtain stable ablation signals in the 200/266 fsLA-ICP-MS (Kimura and Chang, 2012) has been demonstrated to provide signal stability equivalent to that of solution analysis in lead isotope analysis (Chang et al., 2014).

The precision of the 200/266 fsLA-ICP-MS is equivalent at both the 200 nm and 266 nm wavelengths used by this instrument. Reproducibility under matrix-matched conditions was verified through multiple analyses of BCR-2G and BIR-1G using BHVO-2G as the standard material. Results from five replicate measurements showed precision of less than 1.5% for SiO_2_, TiO_2_, Al_2_O_3_, MgO, and CaO, and less than 3.7% for other elements (Kimura and Chang, 2012).

For the particle size distribution of glass standards at 1 Hz per 30 mm crater in this apparatus, the median diameter measured by both 200 nm and 266 nm femtoseconds LA was 115 nm, showing little difference between them. Compared to the 80 nm median diameter of aerosols measured by 193 nm excimer LA, this indicates coarser particles.

***Element incorporation into foraminifera tests***

While the elucidation of element partitioning has been based on core top samples and culture experiments, it has been shown that element partitioning of foraminiferal calcite is not in equilibrium with seawater as defined by inorganic experiments (e.g., de Nooijer et al., 2014). The Mg/Ca ratio of foraminifera tests is likely to be actively decreased due to the active transport pump that acts on the calcifying fluid (Nürnberg et al., 1996; de Nooijer et al., 2014; Bentov, et al., 2006). Recent studies of element distribution within tests at the micro- and nano-scale have revealed that there are high-amplitude Mg/Ca fluctuations that deviate from the temperature calibration formula, and that some of these fluctuations are related to the day/night calcification cycle (Fehrenbacher et al., 2017). In some cases, the Mg/Ca variation within the test corresponds to a 40°C change in reconstructed water temperature (Jonkers et al., 2021). There is a characteristic synchrony and heterogeneity of concentration bands for Mg and other trace elements such as Na, and this has been confirmed in both living species and fossils (Fehrenbacher et al., 2021; Jonkers et al., 2021; Eggins et al., 2004; Sadekov et al., 2005; Spero et al., 2015; Fehrenbacher et al., 2014; John et al., 2023). Several important factors contribute to the variation in element concentrations within the test: changes in the calcifying fluid composition due to cellular processes of the foraminifera themselves or symbiotic algae (de Nooijer et al., 2014; Bentov et al., 2006; Jonkers et al., 2021; Eggins et al., 2004; Spero et al., 2015; Erez et al., 2003); sharp high-concentration zones of both Mg and Na associated with organic templates (Branson et al., 2016; Bonnin et al., 2019; Kunioka et al., 2006). The number, thickness, and arrangement of high-Mg bands differ between species, and that this is the cause of inter-species variation in average elemental composition (Eggins et al., 2004; Sadekov et al., 2005; Spero et al., 2015).

***Foraminifera ecology***

When applying the Mg/Ca thermometer, it is necessary to understand how Mg is distributed within and between the chambers (Kozdon et al., 2011, 2013). Eggins et al. (2003) found that the Mg/Ca ratios of the each chamber of different species, including *T. sacculifer*, corresponded well to temperature changes and foraminiferal life cycles (Bijma and Hemleben, 1994). There has been a concern that measurements on the final chamber of *T. sacculifer* will be biased towards lower Mg/Ca values than the bulk test analysis (Rustic et al., 2021; Sadekov et al., 2005). Similar migration patterns have been recorded in the Mg/Ca values of *Globigerinoides bulloides* in surface sediments, with the final chamber always having the lowest Mg/Ca, and this is interpreted as reflecting changes in deep-sea habitats associated with individual development (Marr et al., 2011).

Most planktonic foraminifera have a large temperature tolerance range of about 14-32°C, and in the case of cultured *T. sacculifer*, 23.5°C is reported to be optimal for chamber formation, gametogenesis, and food reception (Bijma et al., 1990).^66^ The salinity tolerance of *T. sacculifer* in open ocean conditions does not constrain distribution, but salinity indirectly affects the vertical distribution of planktonic foraminifera because it alters vertical mixing and nutrient dynamics in the water column.^66^ Salinity has a minor control on Mg/Ca partitioning (e.g., Lea et al., 1999; Russell et al., 2004). Given the range of salinity change in the water column of sampling site is less than 1 psu (Figure S-1) and that the salinity sensitivity of *T. sacculifer* Mg/Ca is generally low (Dissard et al., 2021), the effect is likely to be small.

The elemental distribution at the microstructural level of Mg is complex. In the cross-sections of the tests of living foraminifera, there are cases where the high concentration bands of other trace elements such as Na are in sync and there are cases where there is a lag (Branson et al., 2016; Bonnin et al., 2019). On the other hand, the potential controlling factors of cross-section fine element patterns may not be clear in average values of the bulk tests or individual chambers (Hauzer et al., 2018). By selectively analyzing the specific chamber of foraminifera *T. sacculifer* collected from surface seawater, a direct comparison of the elemental ratios with the temperature and salinity has been made (Spero et al., 2015; Dissard et al., 2021). In this study, the chemical composition of foraminifera was analyzed in each chamber to reinforce the use of element proxies of foraminifera in paleoceanography, related with test morphology and chamber addition.

Depth migration of *T. sacculifer*, particularly the formation of sac-like final chambers, has been discussed based on plankton nets collected samples in the Eastern Equatorial Atlantic.^1^ The *T. sacculifer* without sac showed a clear abundance peak within the surface mixed layer, while the with-sac form was found just below the mixed layer within the thermocline.^1^ Therefore, a without-sac morphology is often used to avoid bias due to potential depth migration (e.g., Coadic et al., 2013). The δ^18^O measurements of *T. sacculifer* with sac collected from a multicore off northeastern Brazil in the Atlantic Ocean is 0.2 ± 0.4 ‰ (1σ) higher than the previously formed chamber, suggesting sac formation temperature about 1°C lower (Pracht et al., 2019). Although the above results are for the Atlantic Ocean, having a large seasonal thermal structure change, absolute values of calcification depth and temperature vary between and within ocean basins.

The TE/Ca of two different morphology of *T. sacculifer* suggests that sac and/or final calcite layer in with sac samples may be vulnerable to contamination. For *T. sacculifer* with sac, the effect of this contamination is particularly large in the sac-like F0 and F1 chambers. The sac-like final chamber is thinner than the thickened inner chambers (Bé, 1980) and therefore has a larger specific surface area, which can be significantly affected by contaminants on the test surface if they affect the ME/Ca. *T. sacculifer* without sac was reconfirmed to be more suitable for paleoceanographic reconstruction. Note that the discussion in the following sections will not deal with UPW-MeOH cleaned individuals, but only with the elemental ratios of the H_2_O_2_-HClO_4_ oxidation cleaned individuals.

*P. obliquiloculata* distributes in the convergence zone between the South Equatorial Current and the North Equatorial Counter Current. In the equatorial Atlantic, *P. obliquiloculata* is the most abundant near 60 m at the bottom of the seasonal thermocline,^1^ and calcification depths estimated from core-top samples also agree well with 50 m.^80^ Estimated calcification depth of this species obtained from core samples from the West Caroline Basin are 115 ± 20 m, with a habitat temperature of 22.4 ± 2.5°C associated with the base of the upper thermocline (Sagawa et al., 2012). As for calcification temperature of our samples, the Anand et al.'s Atlantic species-specific formula gives a calcification depth of 170-220 m (Anand et al., 2003), which corresponds mainly to the lower thermocline. On the other hand, using the Atlantic mixed-species equation including *P. obliquiloculata* (Anand et al., 2003) and the WPWP (Sagawa et al., 2012), the calcification temperature is about 4-5°C higher than in Anand et al.’s equation.

***Mg/Ca paleotemperature equations***

Several equations for Mg/Ca and temperature for *T. sacculifer* have been proposed. Previous studies closely examined the calibration equation only for *T. sacculifer*, but at the same time, a calibration curve for mixed species also accurately described their temperature dependence (Sagawa et al., 2012; Anand et al., 2003). Comparing calibration curves for individual species, t the pre-exponential and exponential constants of temperature equation, Mg/Ca = B exp(AT), are correlated, and large temperature gradients give small intercept values, so this method has been used to determine the temperature dependence of calibration in a multi-species planktonic foraminifera data set and to describe interspecies differences via the pre-exponential constants (Anand et al., 2003). Table 3 summarizes the typical temperature dependence. The cleaning method used in Dekens et al. (2021) includes a reduction process, which has been reported to result in a 15% lower Mg/Ca value (Rosenthal et al., 2004) than the method consisting of an oxidation process, etc. Therefore, the Mg/Ca value was multiplied by 0.85 to apply for the temperature conversion formula of *T. sacculifer* proposed by Dekens et al. (2002). The differences between the various formulae are due to different conditions, such as plankton nets, surface sediments, and culture experiments, and the statistical differences and biases that have occurred in individual formulae are outside the scope of this article and are detailed in previous studies (e.g., Dissard et al., 2021).

A comparison of mixed-species and *G. menardii* specific temperature conversion equations was also made, similar to that discussed for *T. sacculifer*. The difference between the conversion equations for this species, regardless of the mixed-species and species-specific equations, is small. For F0, the difference between the maximum and the minimum values of the water temperature conversion derived from the five equations is 1°C. The mean value of the calcification temperature of F0 obtained from the mixed-species was 21.3°C with a 1SD for 3 equations of 0.3°C, and the species-specific equations obtained from the Atlantic Ocean and the South China Sea by Regenberg et al. give 21.1°C with a 1SD for 3 equations of 0.7°C (Regenberg et al., 2009, 2010). Although the difference in the choice of equation was small, we adopted the mean value of the species-specific equation, following the same strategy as that of *T. sacculifer*.

**
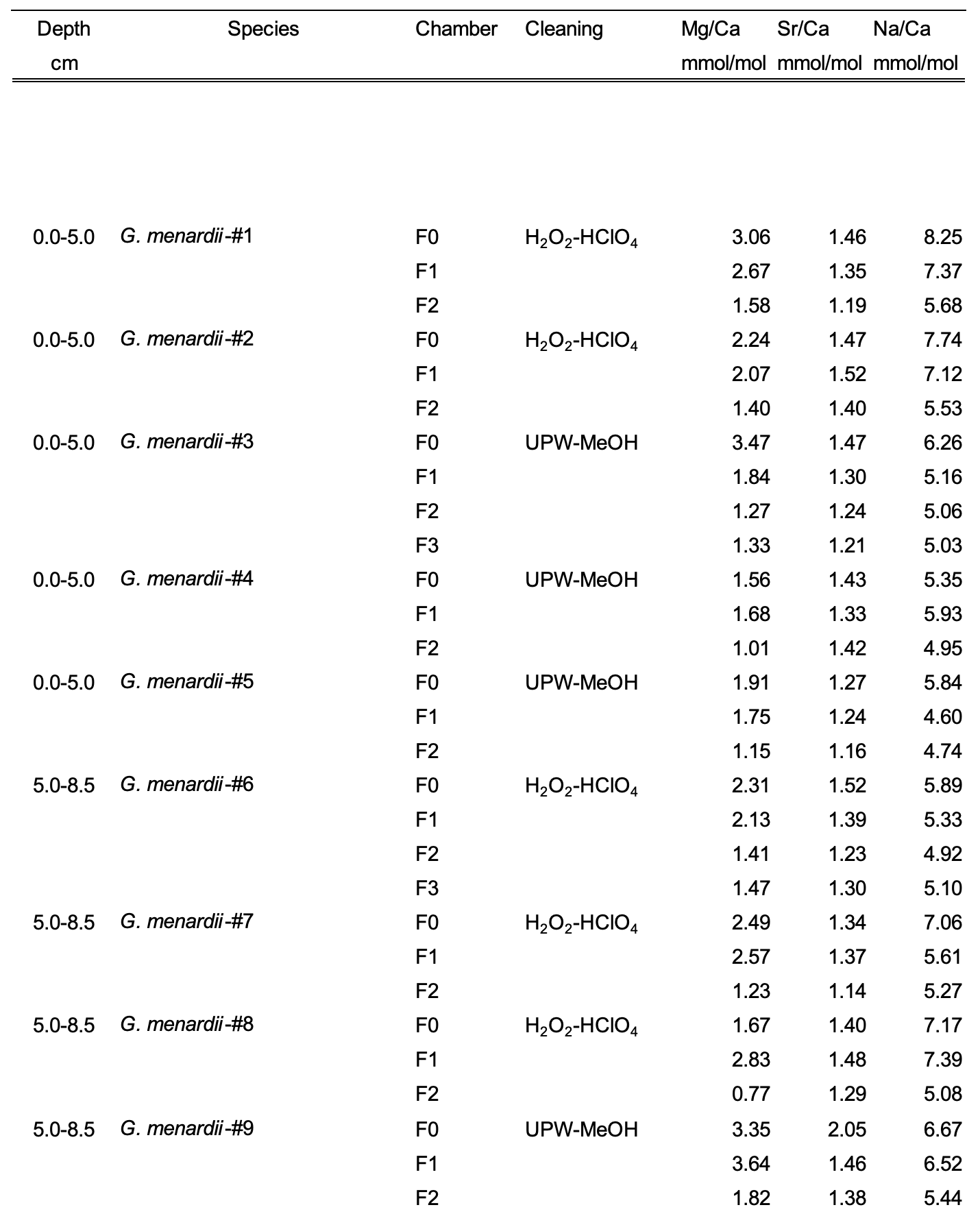
**

**Table S-1.** Data measured for each chamber in *G. menardii*.


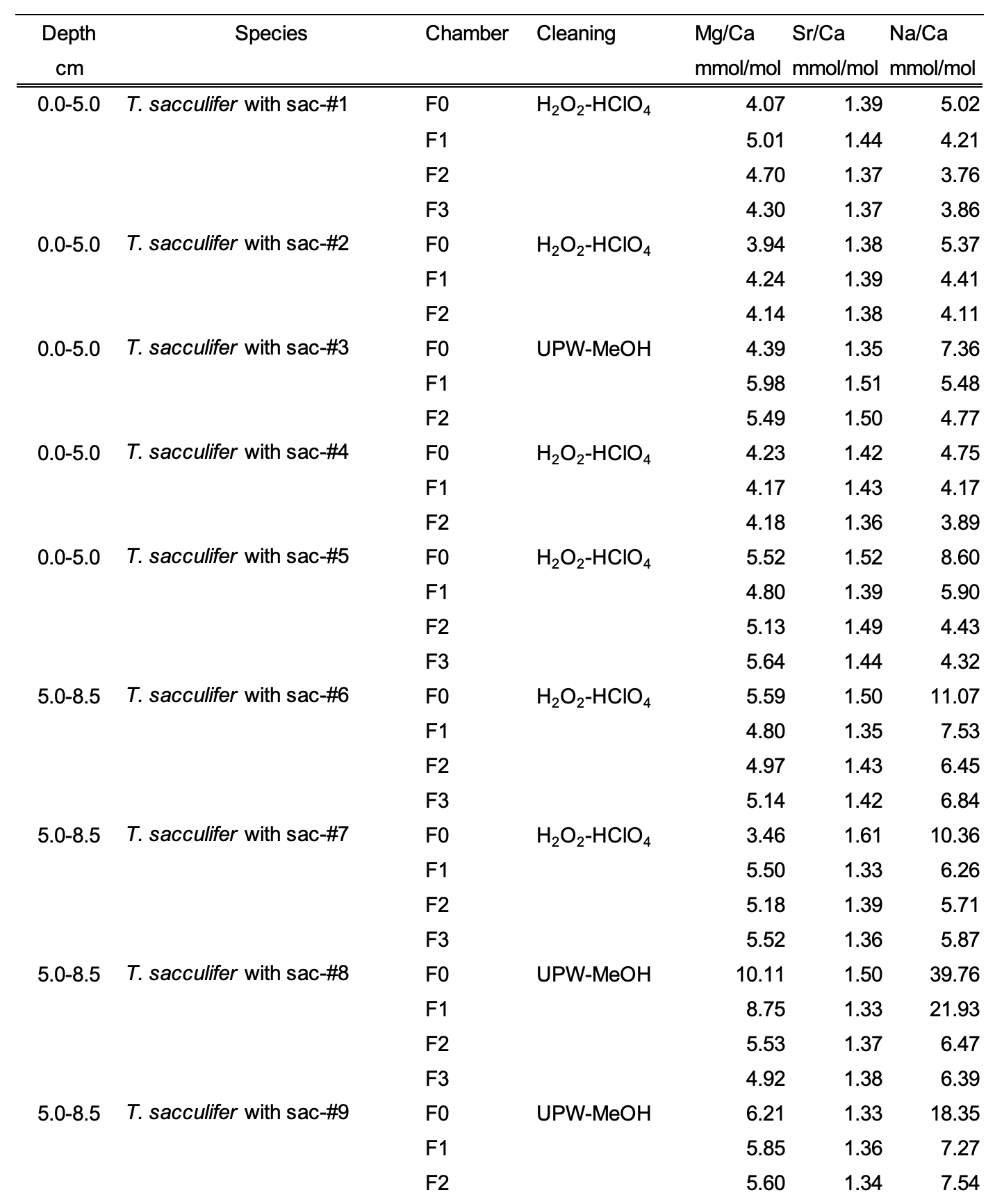


**Table S-2.** Data measured for each chamber in *T*. *sacculifer* with sac.


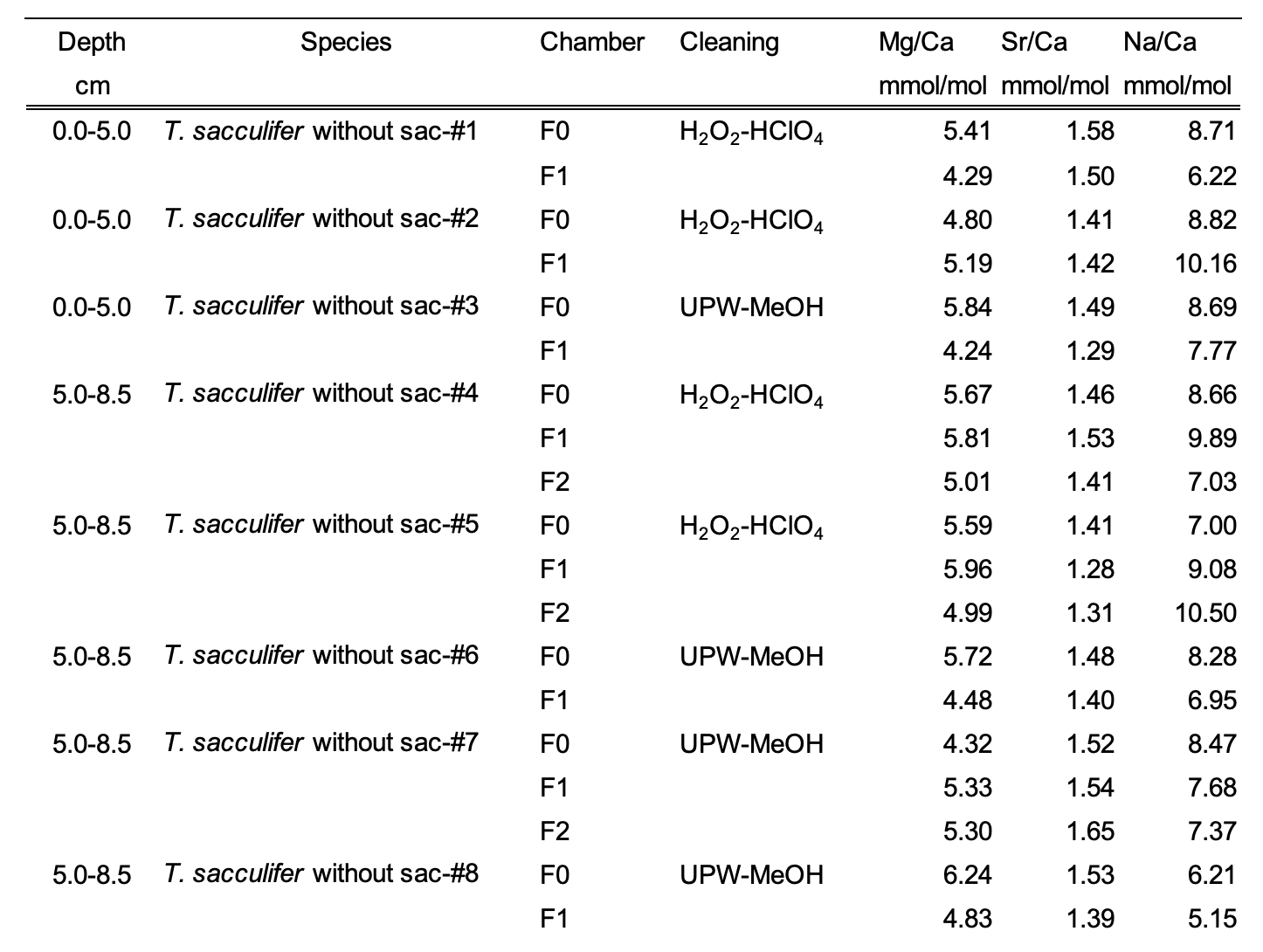


**Table S-3.** Data measured for each chamber in *T*. *sacculifer* without sac.

| Significant |
| --- |
| **#1. H_2_O_2_-HClO_4_ VS UPW-MeOH** |
| Shapiro-Wilk W = 0.79069, p-value = 4.417e-07 |
| F-test F = 0.19281, num df = 31, denom df = 18, p-value = 6.413e-05 |
| Mann-Whitney W = 169.5, p-value = 0.009026 |
| **#2. H_2_O_2_-HClO_4_ with sac VS H_2_O_2_-HClO_4_ without sac** |
| Shapiro-Wilk W = 0.95267, p-value = 0.1715 |
| F-test F = 0.66231, num df = 9, denom df = 21, p-value = 0.5343 |
| Student-t t = 2.337, df = 30, p-value = 0.02631 |
|  |
| Insignificant |
| **#3. H_2_O_2_-HClO_4_ with sac F0 VS H_2_O_2_-HClO_4_ with sac F1, F2, F3** |
| Shapiro-Wilk W = 0.93521, p-value = 0.1575 |
| F-test F = 2.9641, num df = 5, denom df = 15, p-value = 0.09354 |
| Student-t t = -1.2392, df = 20, p-value = 0.2296 |
| **#4. H_2_O_2_-HClO_4_ with sac F1 VS H_2_O_2_-HClO_4_ with sac F0, F2, F3** |
| Shapiro-Wilk W = 0.93521, p-value = 0.1575 |
| F-test F = 0.51579, num df = 5, denom df = 15, p-value = 0.4791 |
| Student-t t = 0.069189, df = 20, p-value = 0.9455 |
| **#5. H_2_O_2_-HClO_4_ with sac F2 VS H_2_O_2_-HClO_4_ with sac F0, F1, F3** |
| Shapiro-Wilk W = 0.93521, p-value = 0.1575 |
| F-test F = 0.4386, num df = 5, denom df = 15, p-value = 0.3705 |
| Student-t t = -0.09338, df = 20, p-value = 0.9265 |
| **#6. H_2_O_2_-HClO_4_ with sac F3 VS H_2_O_2_-HClO_4_ with sac F0, F1, F2** |
| Shapiro-Wilk W = 0.93521, p-value = 0.1575 |
| F-test F = 0.96366, num df = 3, denom df = 17, p-value = 0.8651 |
| Student-t t = 1.4821, df = 20, p-value = 0.1539 |
| **#7. H_2_O_2_-HClO_4_ with sac F0 VS H_2_O_2_-HClO_4_ with sac F1** |
| Shapiro-Wilk W = 0.92941, p-value = 0.3739 |
| F-test F = 3.1469, num df = 5, denom df = 5, p-value = 0.234 |
| Student-t t = -0.69075, df = 10, p-value = 0.5054 |
| **#8. H_2_O_2_-HClO_4_ with sac F0 VS H_2_O_2_-HClO_4_ with sac F2** |
| Shapiro-Wilk W = 0.94019, p-value = 0.5006 |
| F-test F = 3.6201, num df = 5, denom df = 5, p-value = 0.1843 |
| Student-t t = -0.6116, df = 10, p-value = 0.5545 |
| **#9. H_2_O_2_-HClO_4_ with sac F0 VS H_2_O_2_-HClO_4_ with sac F3** |
| Shapiro-Wilk W = 0.86586, p-value = 0.08943 |
| F-test F = 2.1147, num df = 5, denom df = 3, p-value = 0.571 |
| Student-t t = -1.3391, df = 8, p-value = 0.2173 |
| **#10. H_2_O_2_-HClO_4_ with sac F1 VS H_2_O_2_-HClO_4_ with sac F2** |
| Shapiro-Wilk W = 0.91234, p-value = 0.2286 |
| F-test F = 1.1504, num df = 5, denom df = 5, p-value = 0.8816 |
| Student-t t = -0.13236, df = 10, p-value = 0.8973 |
| **#11. H_2_O_2_-HClO_4_ with sac F1 VS H_2_O_2_-HClO_4_ with sac F3** |
| Shapiro-Wilk W = 0.91209, p-value = 0.2957 |
| F-test F = 0.67199, num df = 5, denom df = 3, p-value = 0.6496 |
| Student-t t = 1.1384, df = 8, p-value = 0.2879 |
| **#12. H_2_O_2_-HClO_4_ with sac F2 VS H_2_O_2_-HClO_4_ with sac F3** |
| Shapiro-Wilk W = 0.91725, p-value = 0.3346 |
| F-test F = 0.58414, num df = 5, denom df = 3, p-value = 0.5586 |
| Student-t t = 1.2889, df = 8, p-value = 0.2334 |
| **#13. H_2_O_2_-HClO_4_ without sac F0 VS H_2_O_2_-HClO_4_ without sac F1, F2** |
| Shapiro-Wilk W = 0.96449, p-value = 0.8356 |
| F-test F = 0.41707, num df = 3, denom df = 5, p-value = 0.5026 |
| Student-t t = 0.45764, df = 8, p-value = 0.6594 |
| **#14. H_2_O_2_-HClO_4_ without sac F1 VS H_2_O_2_-HClO_4_ without sac F0, F2** |
| Shapiro-Wilk W = 0.96449, p-value = 0.8356 |
| F-test F = 4.4622, num df = 3, denom df = 5, p-value = 0.1409 |
| Student-t t = 0.19203, df = 8, p-value = 0.8525 |
| **#15. H_2_O_2_-HClO_4_ without sac F2 VS H_2_O_2_-HClO_4_ without sac F0, F1** |
| Shapiro-Wilk W = 0.96449, p-value = 0.8356 |
| F-test F = 0.00063688, num df = 1, denom df = 7, p-value = 0.03886 |
| Welch-t t = 1.7139, df = 7.0354, p-value = 0.1301 |
| **#16. H_2_O_2_-HClO_4_ without sac F0 VS H_2_O_2_-HClO_4_ without sac F1** |
| Shapiro-Wilk W = 0.92265, p-value = 0.4517 |
| F-test F = 0.26914, num df = 3, denom df = 3, p-value = 0.3096 |
| Student-t t = 0.12868, df = 6, p-value = 0.9018 |
| **#17. H_2_O_2_-HClO_4_ without sac F0 VS H_2_O_2_-HClO_4_ without sac F2** |
| Shapiro-Wilk W = 0.90265, p-value = 0.3898 |
| F-test F = 774.79, num df = 3, denom df = 1, p-value = 0.0528 |
| Student-t t = 1.2445, df = 4, p-value = 0.2813 |
| **#18. H_2_O_2_-HClO_4_ without sac F1 VS H_2_O_2_-HClO_4_ without sac F2** |
| Shapiro-Wilk W = 0.9377, p-value = 0.6408 |
| F-test F = 2878.8, num df = 3, denom df = 1, p-value = 0.0274 |
| Welch-t t = 0.8234, df = 3.0042, p-value = 0.4706 |
|  |
| ANOVA |
| **#19. H_2_O_2_-HClO_4_ with sac，ANOVA** |
| Shapiro-Wilk W = 0.93521, p-value = 0.1575 |
| Bartlet K-squared = 2.4562, df = 3, p-value = 0.4833 |
| One-way ANOVA (equal) Sum_Sq = 1.19, df = 3, F = 0.923, p-value = 0.4497 |
| **#20. H_2_O_2_-HClO_4_ without sac, ANOVA** |
| Shapiro-Wilk W = 0.96449, p-value = 0.8356 |
| Bartlet Bartlett's K-squared = 6.0932, df = 2, p-value = 0.04752 |
| One-way ANOVA (unequal) F = 1.7799, num df = 2.000, denom df = 4.013, p-value = 0.2797 |

**Table S-4.** Results of statistical analysis examining differences in Mg/Ca in *T. sacculifer* by morphology and chamber

**Table S-5.** Elemental composition of cleaning supernatant. Two size fractions (63-150, 150-250 μm) of sediment samples from the Ontong Java Plateau were cleaned with reagents after removing fine-grained material other than foraminifera using ultrapure water and methanol. The cleaning sequence was performed twice with a mixture of ammonium hydroxide, sodium hydroxide, potassium hydroxide, and hydrogen peroxide, followed by one treatment with a mixture of perchloric acid and hydrogen peroxide.

**
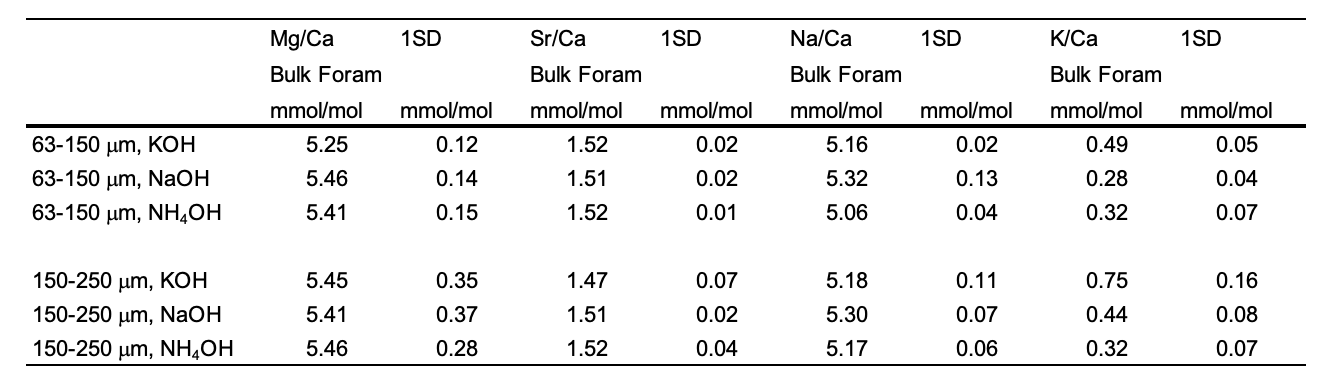
**

**Table S-6.** Ratios of Mg/Ca, Sr/Ca, Na/Ca, and K/Ca in residual foraminifera shells obtained after oxidation cleaning. Two grain size fractions (63–150 μm and 150–250 μm) were extracted from the same sediment sample used for laser analysis. For each sample, 4 mg of foraminifera shells were subjected to sequential cleaning without species sorting.


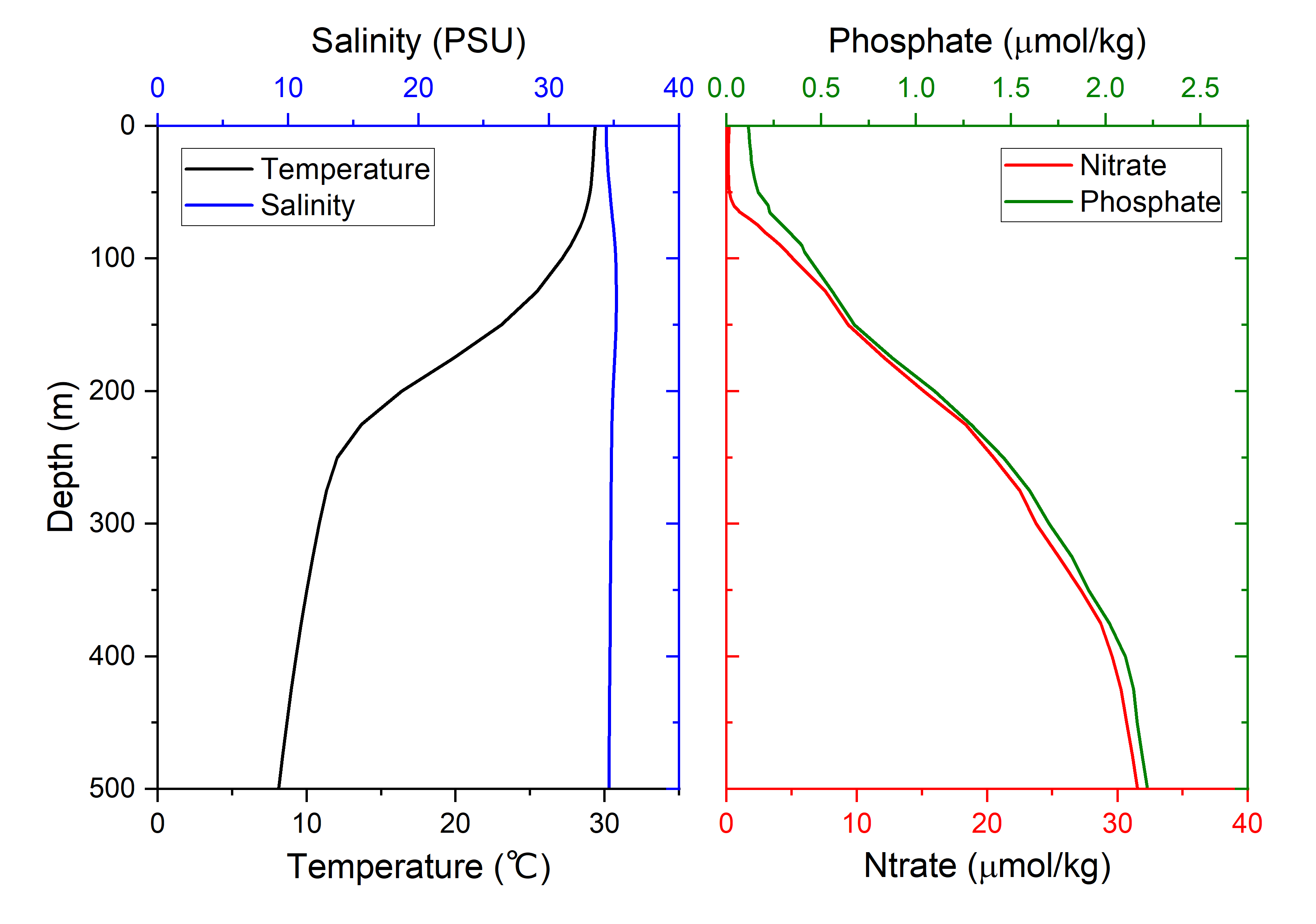


**Figure S-1.** Vertical profiles of water temperature, salinity, and nutrient concentrations (nitrate and phosphate) at the WOA site near the sample collection site.


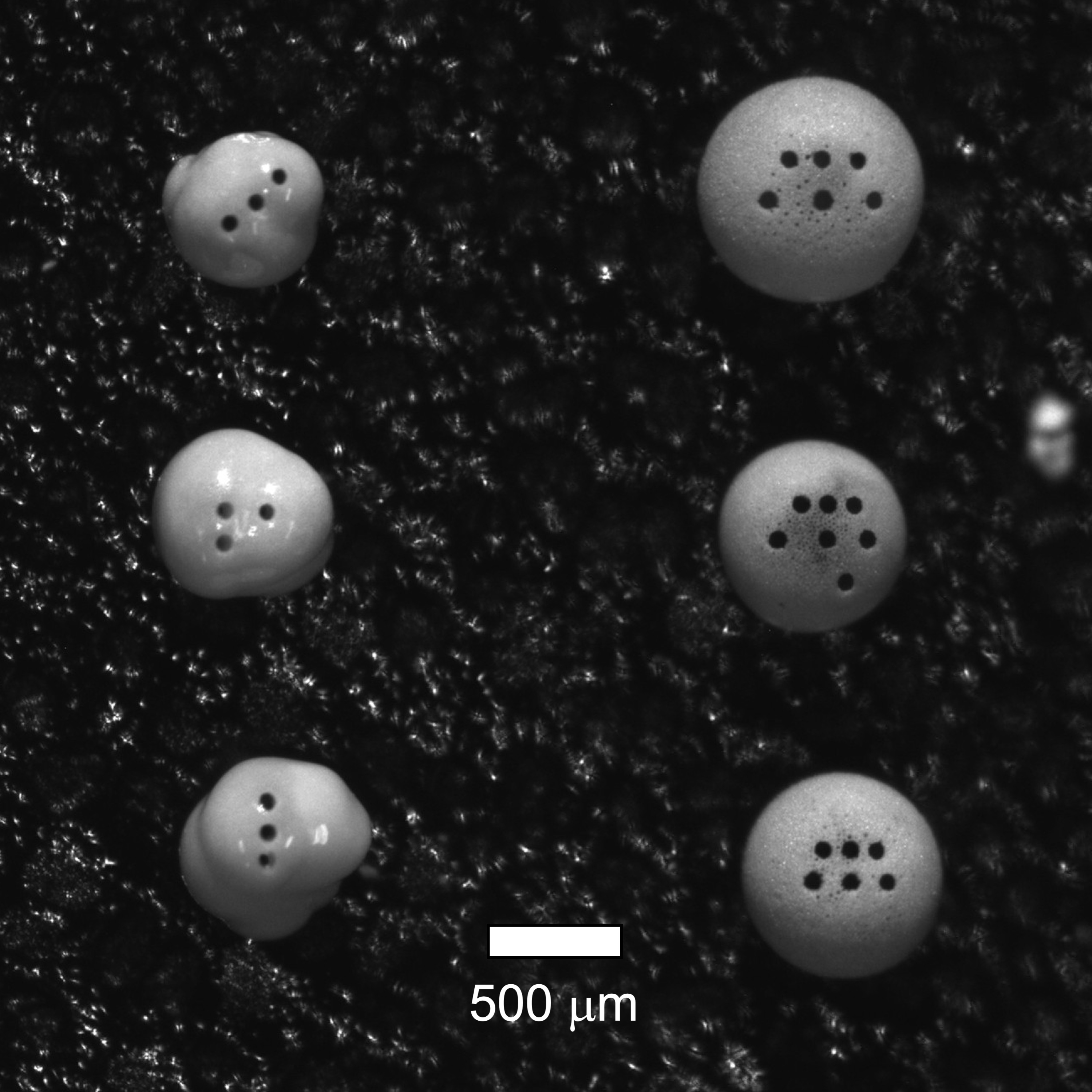


**Figure S-2**. Examples of laser-analyzed individual samples. The laser sampling spots of *O. universa* (right) and *P. obliquiloculata* (left) used in the repeated measurements are generally around 40-60 μm in size.


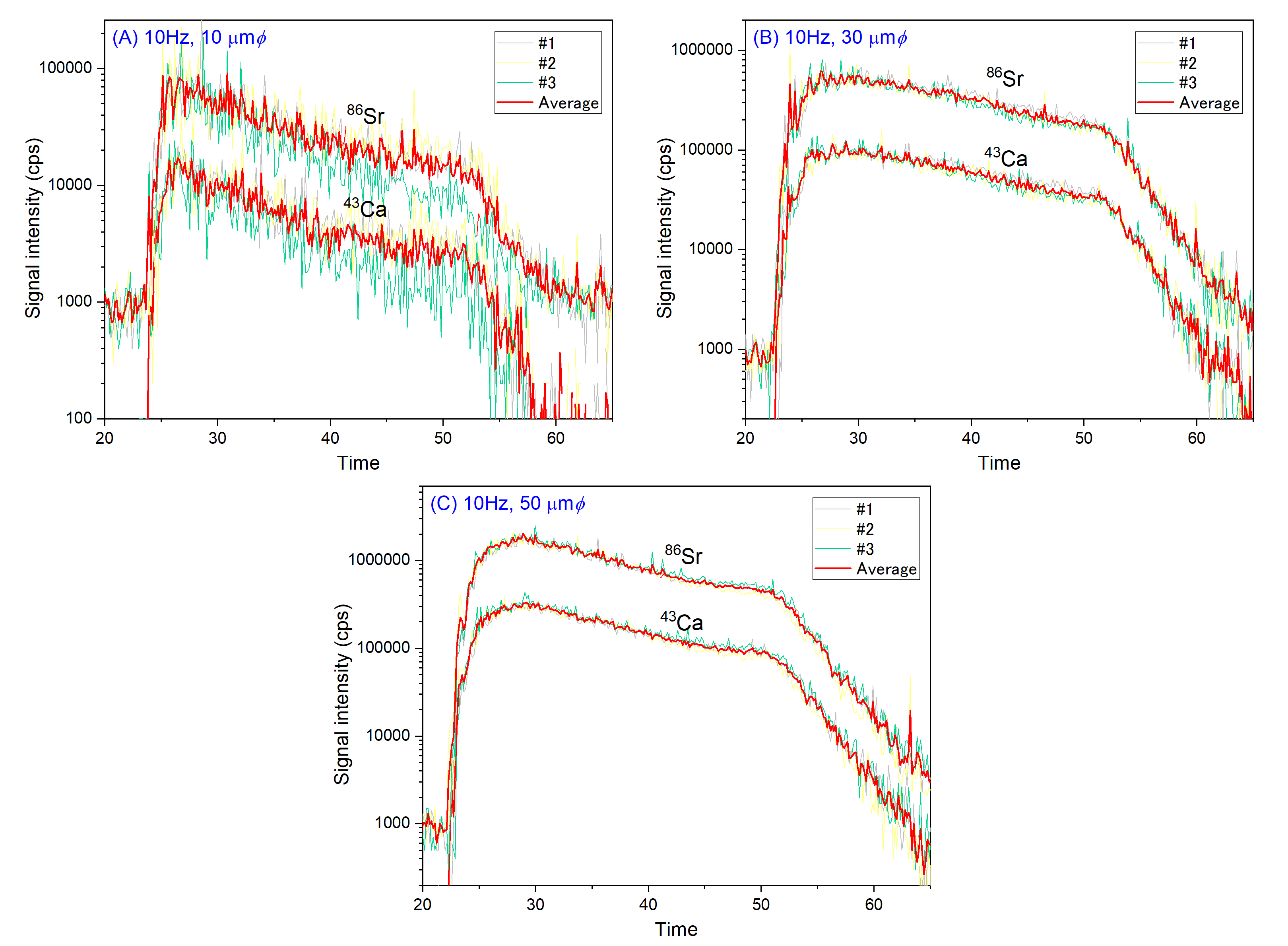


**Figure S-3**. Experiments in LA-ICP-MS analysis with a fixed repetition rate of 10 Hz and laser sizes of 10, 30, and 50 μm. Three repetitions of each condition are shown for the JCp-1 sample, and the average values are shown. Larger laser diameters provide stronger signal intensity and thus improve the signal-to-noise ratio of the measurements.


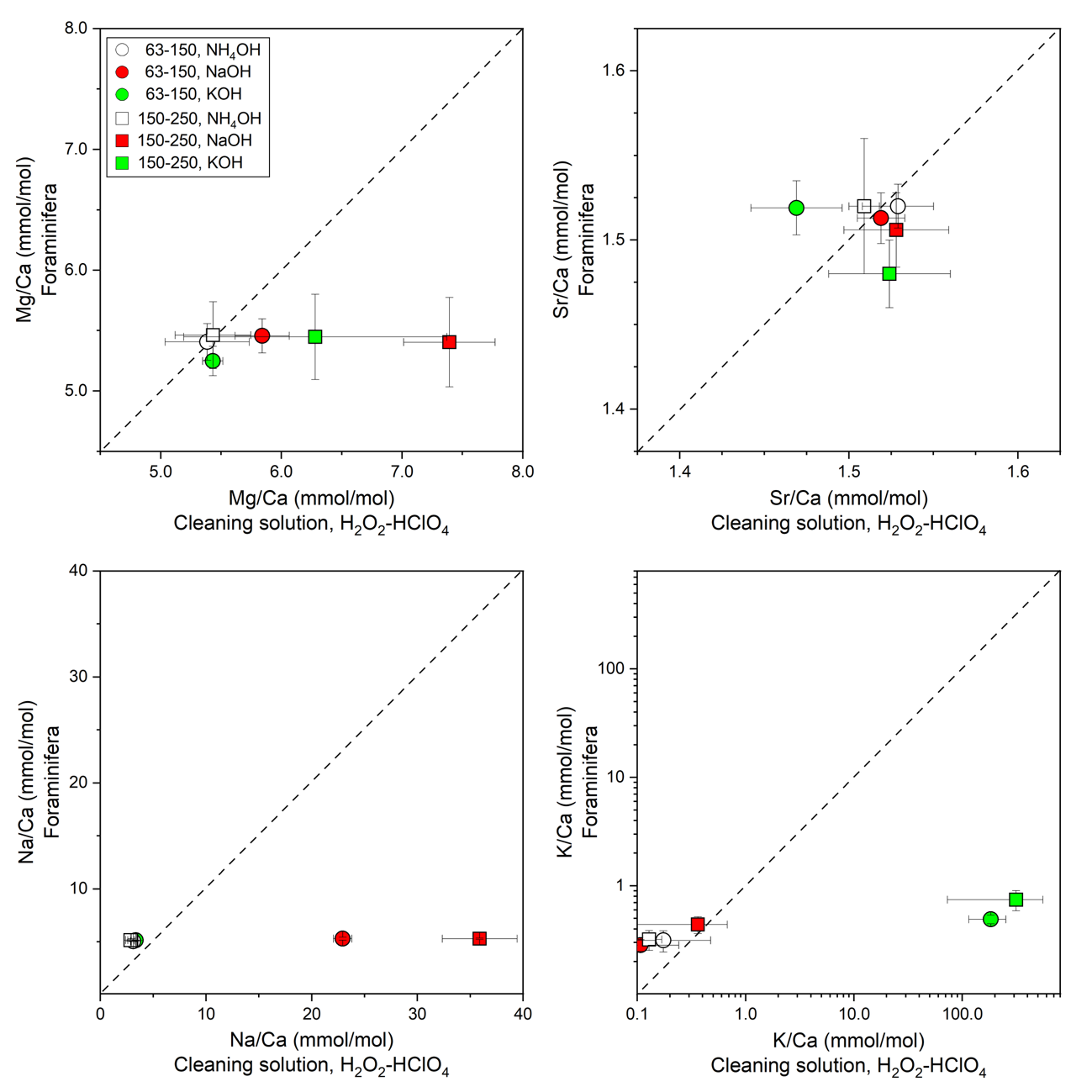


**Figure S-4**. Comparison of Mg/Ca, Sr/Ca, Na/Ca, and K/Ca ratios in residual foraminifera shells obtained after oxidation cleaning and in the second cleaning solution (H_2_O_2_-HClO_4_). A dotted line at 1:1 indicates the effect of stoichiometric partial dissolution of the foraminifera shells for comparison. While Mg/Ca in most samples and Sr/Ca in nearly all samples are close to the composition of partially dissolved shells, Na/Ca and K/Ca show carryover of NaOH and KOH from the initial cleaning reagent.


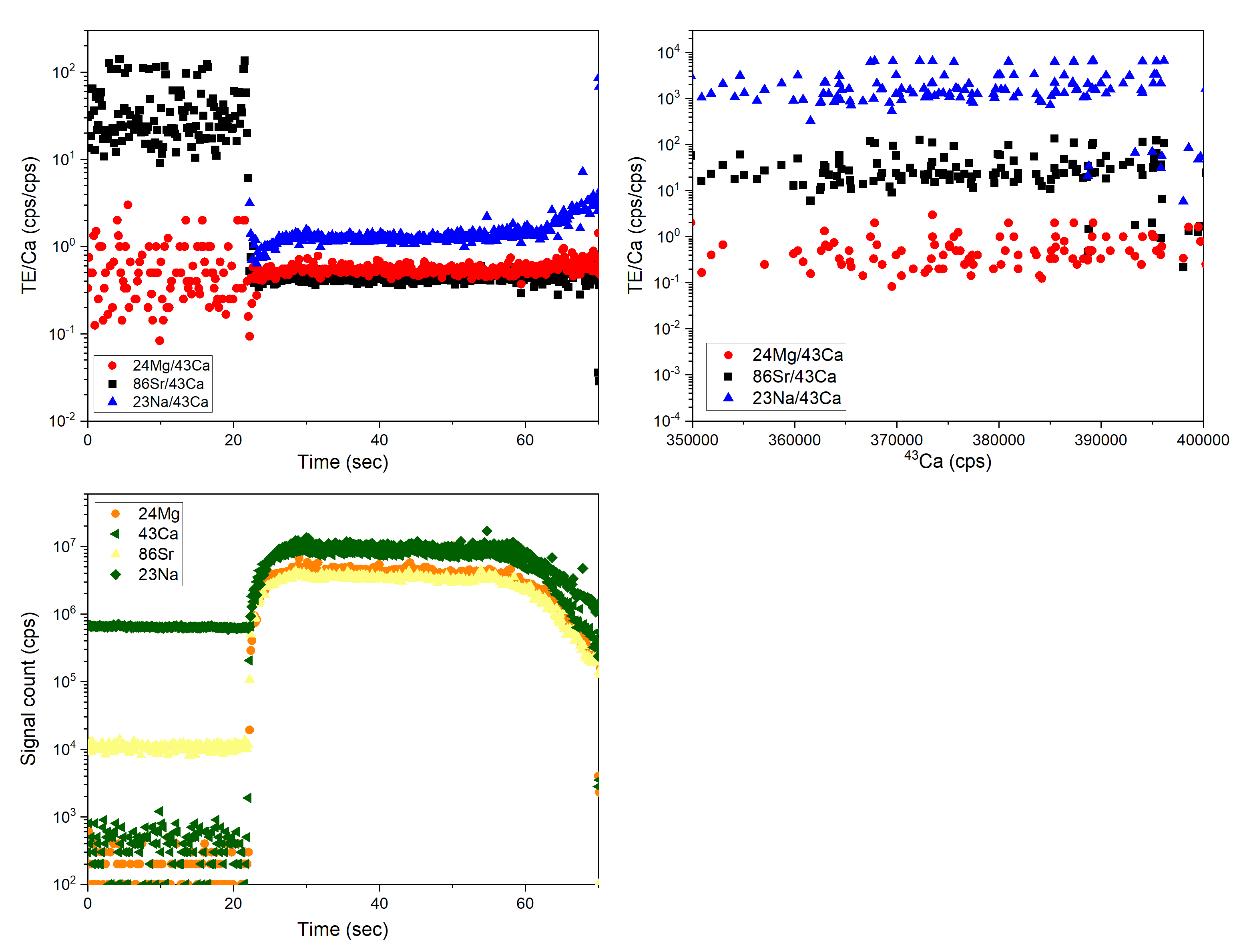


PL04, 0-5 cm

*T. sacculifer* with sac, F1


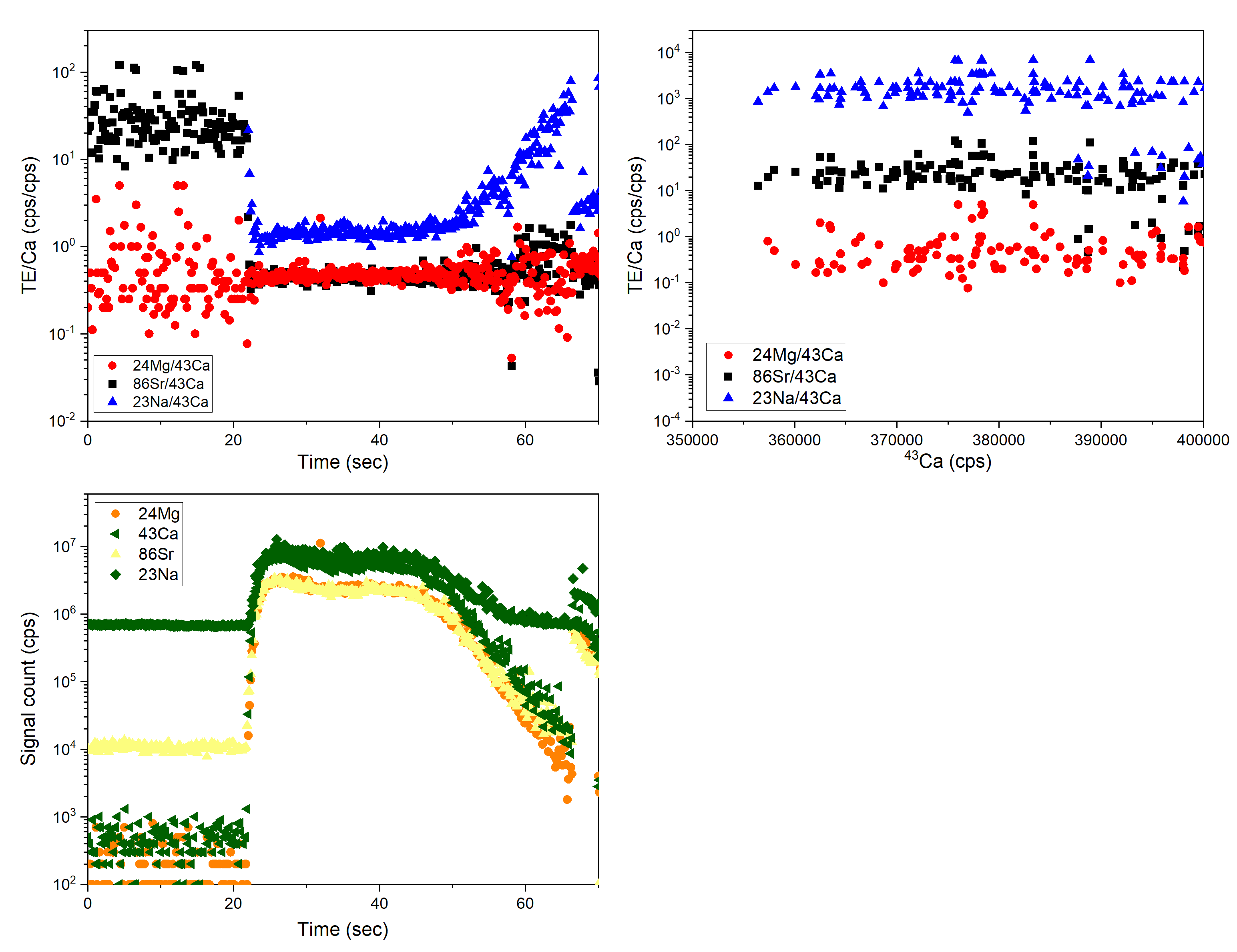


PL04, 0-5 cm

*T. sacculifer* with sac, F0

**Figure S-5**. Two example of raw measurement data of *T. sacculifer* F1 (upper) and F0 chamber (lower) picked from 0-5 cm of the core. Each upper left panel shows the ratio of signal intensity for the target element to measurement time on the horizontal axis; lower left panel shows signal intensity versus time on the horizontal axis; upper right panel shows calcium signal intensity on the horizontal axis and signal intensity ratio on the vertical axis. Element ratios were determined during the calcium intensity plateau section. The section where signal intensity began to decrease indicates completion of the shell depth profile measurement and was not used for integration. In the region where calcium signal intensity is strong, stable element ratio signals can be obtained independently of the ^43^Ca signal intensity. The F1 shell is thicker than the F0 shell, resulting in a longer ablation time on the horizontal axis and yielding a smoothed depth profile via rotation raster mode.

**References**

Anand, P.; Elderfield, H.; Conte, M. H. Calibration of Mg/Ca thermometry in planktonic foraminifera from a sediment trap time series. *Paleoceanography* **2003**, *18*, 1050.

Bé, A. Gametogenic calcification in a spinose planktonic foraminifer, *Globigerinoides sacculifer* (Brady). *Mar. Micropaleontol.***1980**, *5*, 283-310.

Bentov, S.; Erez, J., Impact of biomineralization processes on the Mg content of foraminiferal shells: A biological perspective. *Geochem. Geophy. Geosys.* **2006**, *7*, Q01P08.

Bijma, J.; Hemleben, C., Population dynamics of the planktic foraminifer Globigerinoides sacculifer (Brady) from the central Red Sea. *Deep Sea Res. I* **1994,** *41* (3), 485-510.

Bijma, J.; Faber, W. W.; Hemleben, C. Temperature and salinity limits for growth and survival of some planktonic foraminifers in laboratory cultures. *J. Foram. Res.* **1990**, *20*, 95-116.

Bonnin, E. A.; Zhu, Z.; Fehrenbacher, J. S.; Russell, A. D.; Hönisch, B.; Spero, H. J.; Gagnon, A. C., Submicron sodium banding in cultured planktic foraminifera shells. *Geochim. Cosmochim. Acta* **2019,** *253*, 127-141.

Branson, O.; Bonnin, E. A.; Perea, D. E.; Spero, H. J.; Zhu, Z.; Winters, M.; Hönisch, B.; Russell, A. D.; Fehrenbacher, J. S.; Gagnon, A. C., Nanometer-scale chemistry of a calcite biomineralization template: Implications for skeletal composition and nucleation. *Proc. Natl. Acad. Sci.* **2016,** *113*, 12934-12939.

Coadic, R.; Bassinot, F.; Dissard, D.; Douville, E.; Greaves, M.; Michel, E. A core‐top study of dissolution effect on B/Ca in *Globigerinoides sacculifer* from the tropical Atlantic: Potential bias for paleo‐reconstruction of seawater carbonate chemistry. *Geochem. Geophys. Geosys.* **2013**, *14*, 1053-1068.

Dekens, P. S.; Lea, D. W.; Pak, D. K.; Spero, H. J. Core top calibration of Mg/Ca in tropical foraminifera: Refining paleotemperature estimation. *Geochem. Geophys. Geosys.* **2002**, *3*, 1-29.

de Nooijer, L. J.; Spero, H. J.; Erez, J.; Bijma, J.; Reichart, G.-J., Biomineralization in perforate foraminifera. *Earth-Sci. Rev.* **2014**, 135, 48-58.

Dissard, D.; Reichart, G. J.; Menkes, C.; Mangeas, M.; Frickenhaus, S.; Bijma, J., Mg∕ Ca, Sr∕ Ca and stable isotopes from the planktonic foraminifera T. sacculifer: testing a multi-proxy approach for inferring paleotemperature and paleosalinity. *Biogeosciences* **2021,** *18*, 423-439.

Eggins, S.; De Deckker, P.; Marshall, J. Mg/Ca variation in planktonic foraminifera tests: implications for reconstructing palaeo-seawater temperature and habitat migration. *Earth Planet. Sci. Lett.* **2003**, *212*, 291-306.

Eggins, S.; Sadekov, A.; Dedeckker, P. Modulation and daily banding of Mg/Ca in tests by symbiont photosynthesis and respiration: a complication for seawater thermometry? *Earth Planet. Sci. Lett.* **2004**, *225*, 411-419.

Erez, J., The source of ions for biomineralization in foraminifera and their implications for paleoceanographic proxies. *Rev. Mineral. Geochem.* **2003,** *54*, 115-149.

Fehrenbacher, J. S.; Martin, P. A., Exploring the dissolution effect on the intrashell Mg/Ca variability of the planktic foraminifer *Globigerinoides ruber.* *Paleoceanography* **2014,** *29*, 854-868.

Fehrenbacher, J. S.; Russell, A. D.; Davis, C. V.; Gagnon, A. C.; Spero, H. J.; Cliff, J. B.; Zhu, Z.; Martin, P., Link between light-triggered Mg-banding and chamber formation in the planktic foraminifera Neogloboquadrina dutertrei. *Nat. Commun.* **2017**, *8*, 15441.

Hauzer, H.; Evans, D.; Müller, W.; Rosenthal, Y.; Erez, J. Calibration of Na partitioning in the calcitic foraminifer *Operculina ammonoides* under variable Ca concentration: Toward reconstructing past seawater composition. *Earth Planet. Sci. Lett.* **2018**, *497*, 80-91.

John, E. H.; Staudigel, P. T.; Buse, B.; Lear, C. H.; Pearson, P. N.; Slater, S. M., Revealing their true stripes: Mg/Ca banding in the Paleogene planktonic foraminifera genus Morozovella and implications for paleothermometry. *Paleoceanogr. Paleoclimatol.* **2023,** *38*, e2023PA004652.

Jonkers, L.; Brummer, G.-J. A.; Meilland, J.; Groeneveld, J.; Kucera, M., Variability in Neogloboquadrina pachyderma stable isotope ratios from isothermal conditions: implications for individual foraminifera analysis. *Climate Past* **2021**, *18*, 89-101.

Kozdon, R.; Kelly, D. C.; Kita, N. T.; Fournelle, J. H.; Valley, J. W., Planktonic foraminiferal oxygen isotope analysis by ion microprobe technique suggests warm tropical sea surface temperatures during the Early Paleogene. *Paleoceanography* **2011,** *26*, PA3206.

Kozdon, R.; Kelly, D.; Kitajima, K.; Strickland, A.; Fournelle, J.; Valley, J., In situ δ^18^O and Mg/Ca analyses of diagenetic and planktic foraminiferal calcite preserved in a deep‐sea record of the Paleocene‐Eocene thermal maximum. *Paleoceanography* **2013,** *28*, 517-528.

Kunioka, D.; Shirai, K.; Takahata, N.; Sano, Y.; Toyofuku, T.; Ujiie, Y., Microdistribution of Mg/Ca, Sr/Ca, and Ba/Ca ratios inPulleniatina obliquiloculatatest by using a NanoSIMS: Implication for the vital effect mechanism. *Geochem. Geophys. Geosys.* **2006,** *7*, Q12P20.

Lea, D. W.; Mashiotta, T. A.; Spero, H. J., Controls on magnesium and strontium uptake in planktonic foraminifera determined by live culturing. *Geochim. Cosmochim. Acta* **1999,** *63*, 2369-2379.

Marr, J. P.; Baker, J. A.; Carter, L.; Allan, A. S.; Dunbar, G. B.; Bostock, H. C., Ecological and temperature controls on Mg/Ca ratios of *Globigerina bulloides* from the southwest Pacific Ocean. *Paleoceanography* **2011**, 26, PA2209.

Nürnberg, D.; Bijma, J.; Hemleben, C., Assessing the reliability of magnesium in foraminiferal calcite as a proxy for water mass temperatures. *Geochim. Cosmochim. Acta* **1996**, 60, 803-814.

Pracht, H.; Metcalfe, B.; Peeters, F. J. Oxygen isotope composition of the final chamber of planktic foraminifera provides evidence of vertical migration and depth-integrated growth. *Biogeosciences* **2019**, *16*, 643-661.

Regenberg, M.; Nielsen, S. N.; Kuhnt, W.; Holbourn, A.; Garbe-Schönberg, D.; Andersen, N. Morphological, geochemical, and ecological differences of the extant menardiform planktonic foraminifera *Globorotalia menardii* and *Globorotalia cultrata*. *Mar. Micropaleontol.* **2010**, *74*, 96-107.

Regenberg, M.; Steph, S.; Nürnberg, D.; Tiedemann, R.; Garbe-Schönberg, D. Calibrating Mg/Ca ratios of multiple planktonic foraminiferal species with δ18O-calcification temperatures: Paleothermometry for the upper water column. *Earth Planet. Sci. Lett.* **2009**, *278*, 324-336.

Rosenthal, Y.; Perron‐Cashman, S.; Lear, C. H.; Bard, E.; Barker S.; Billups, K.; Bryan, M.; Delaney, M. L.; de Menocal, P. B.; Dwyer, G. S. Interlaboratory comparison study of Mg/Ca and Sr/Ca measurements in planktonic foraminifera for paleoceanographic research. *Geochem. Geophys. Geosys.* **2004**, *5*, Q04D09.

Russell, A. D.; Hönisch, B.; Spero, H. J.; Lea, D. W., Effects of seawater carbonate ion concentration and temperature on shell U, Mg, and Sr in cultured planktonic foraminifera. *Geochim. Cosmochim. Acta* **2004,** *68*, 4347-4361.

Rustic, G. T.; Polissar, P. J.; Ravelo, A. C.; de Menocal, P. Relationship between individual chamber and whole shell Mg/Ca ratios in *Trilobatus sacculifer* and implications for individual foraminifera palaeoceanographic reconstructions. *Sci. Rep.* **2021**, *11*, 463.

Sagawa, T.; Yokoyama, Y.; Ikehara, M.; Kuwae, M. Shoaling of the western equatorial Pacific thermocline during the last glacial maximum inferred from multispecies temperature reconstruction of planktonic foraminifera. 2012, *Palaeogeogr. Palaeoclimatol. Palaeoecol.* **2012**, *346*, 120-129.

Sadekov, A. Y.; Eggins, S. M.; De Deckker, P., Characterization of Mg/Ca distributions in planktonic foraminifera species by electron microprobe mapping. *Geochem. Geophys. Geosys.* **2005**, 6, Q12P06.

Spero, H. J.; Eggins, S. M.; Russell, A. D.; Vetter, L.; Kilburn, M. R.; Hönisch, B., Timing and mechanism for intratest Mg/Ca variability in a living planktic foraminifer. *Earth Planet. Sci. Lett.* **2015,** *409*, 32-42.
